# Communication of children’s weight status: what is effective and what are the children’s and parents’ experiences and preferences? A mixed methods systematic review

**DOI:** 10.1186/s12889-020-08682-w

**Published:** 2020-04-28

**Authors:** H. Ames, A. Mosdøl, N. Blaasvær, H. Nøkleby, R. C. Berg, L. J. Langøien

**Affiliations:** The Norwegian Institute of Public Health, Oslo, Norway

**Keywords:** Communication, Weight, Weight assessment, Weight notification, Children, Adolescents, Parents, Systematic review

## Abstract

**Background:**

Early intervention and conversation about a child’s weight may offer an important chance of success in reducing weight and implementing a healthier lifestyle. This review explores the most effective ways to notify parents and children about the child’s weight as well as their preferences and experiences around weight notification.

**Methods:**

We systematically searched nine databases for relevant primary research. Records were independently screened by two authors. We extracted data into a form designed for this review. Effect data was analysed using narrative synthesis and qualitative data using a best-fit framework synthesis. We assessed our confidence in the evidence using GRADE and GRADE-CERQual.

**Results:**

Studies of effect found that the format of feedback made little or no difference in parents attending further treatment, recognising their child as overweight or obese, reactions to the way the weight notification is given, motivation for lifestyle change, understanding how to reduce the risk of overweight, or taking any action. However, parents receiving feedback with motivational interviewing have somewhat greater satisfaction with the way the healthcare provider supports them.

Qualitative studies found that parents had clear preferences for the format, timing, content and amount of information they wanted to receive in relation to both the weighing process and weight notification. They also had clear preferences for how they wanted health care providers to interact and communicate with them and their children. Both parents and children often felt that they were not receiving enough information and worried about how their results would be kept private. Many parents experienced an emotional response when told about their child’s weight ranging from positive, disbelief and negative feelings. Those who reacted with disbelief or negatively were less likely to accept their child’s weight status and/or act upon the notification letter.

No studies reported results for children who were underweight.

**Conclusions:**

Based on these qualitative results people working with weight assessment and notification programs should consider parents’ preferences when developing feedback formats, considering the mode of feedback they are going to use and provide parents and children with tailored feedback and personalized follow up once a child is identified as overweight or obese.

## Background

Childhood under- and overweight are serious threats to public health in the twenty-first century [1]. Underweight, is a weight considered too low to be healthy, while overweight and obesity are understood as abnormal or excessive fat accumulation that represents a risk to health. Internationally, there is consensus that body mass index (BMI) is the best available anthropometric measurement to identify overweight and obesity among older children, adolescents and adults on a population level [2, 3]. On an individual level, however, BMI cannot distinguish between the relative proportion of fat and muscle mass, nor the body fat distribution [2, 3]. Although the definitions of underweight, overweight and obese vary somewhat between countries, they are generally based on cut-off values (outer percentiles or standard deviation (Z)-scores) related to growth reference charts of weight for age, length/height for weight or BMI-reference curves [4].

Globally, the prevalence of underweight children is decreasing, but remains a problem in many low and middle-income countries [1]. Conversely, the number of obese children and adolescents is today ten times higher than it was 40 years ago, with accelerating trends particularly in low- and middle-income countries [1]. In several European countries, the proportion of overweight or obese children has stabilized in the last ten years, however, about 16% of Norwegian children aged 8–9 years are still overweight or obese [5].

Overweight and obesity in childhood, particularly when present into teenage years, tend to follow a trajectory of overweight and obesity in adulthood [6], with a subsequent higher risk of non-communicable diseases like diabetes and cardiovascular diseases at a young age [7–9]. Thus, childhood obesity has long-term implications for the capacity and costs of healthcare systems [6–9]. The prevalence of underweight children is decreasing, but is still a problem in many low and middle-income countries [1]. Being underweight can have serious long term psychological and health related impacts as well as effect learning abilities [9].

Most countries have healthcare services for monitoring, vaccinations, health education and advice for parents of babies and small children, such as health centres, primary care clinics or well-baby clinics. Supported by WHO recommendations on child health programs, most include routine height- and weight monitoring of babies and small children [10]. The WHO guideline recommends a consultation schedule with regular weighing and measurements of length (0–2 years) or height (> 2 years). In the youngest age groups, children are usually measured at primary health centres with parents present. These repeat consultations offer an opportunity to both healthcare professionals and parents to raise issues of concern, including issues related to the child’s weight status. In fact, health professionals have a duty to follow-up on concerns they identify during consultations, and are in a unique position to support and influence parents in creating a healthy childhood environment [11]. As the child reaches school age, however, when, how and even whether their weight and height are monitored vary significantly between countries. In some countries, monitoring is continued through the school health services [12–14].

The framework for preventive weight monitoring, health education and advice for children and their parents about weight, nutrition and lifestyle is well established. However, reports from different countries show that a considerable proportion of health personnel are uncomfortable with talking about a child’s weight status [15, 16]. Reasons include the sensitive nature of weight in many cultures, fear of doing harm (eating disorders or psychological harm), uncertainties about the cut-offs, lack of communication skills concerning weight and/or uncertainties about what to recommend parents as effective strategies to address the child’s weight problem [15–24]. Further, the effect of weight monitoring on the child’s further weight development can be questioned [25]. Parents’ knowledge about the presence of under- or overweight in itself, even if combined with a conversation with health personnel, may not be enough to trigger actual behavioural changes necessary to change the child’s weight development. Specifically, this will not occur if parents do not perceive that their child is overweight [26]. Several studies have shown that parents of overweight and obese children have inaccurate perceptions of the weight status of their children and often underestimated their weight [27, 28].

Early intervention and conversation about a child’s weight status may increase the chances of reducing weight and implementing a healthier lifestyle [29]. This systematic review focuses on the evidence in this regard, specifically, communication methods and strategies to inform parents and/or the child that routine weight screening results identified that the child was underweight, overweight or obese. We had two research objectives. The first concerned the effect of different communication methods and strategies delivered by health personnel to inform about weight status as compared to usual care or relative to another method/strategy. In the second research objective, we explored parents’ and children’s preferences for and experiences with communication about weight issues as part of routine weight screening and notification programs. This systematic review is based on a 2019 report from the Norwegian Institute of Public Health commissioned as part of a guidelines process by the Norwegian Directorate of Health [30].

## Methods

### Inclusion criteria

The inclusion criteria are listed in Table 1.

**Table 1:** Study inclusion criteria

	Studies of effect (Controlled studies)	Studies of perception and experience (Qualitative studies
**Population:**	Children and parents of children aged 0–19 years.	
**Context:**	Primary health centres, school health programs or similar health-services for preventive monitoring and care. Any country.	
**Intervention/Topic of interest:**	Any intervention using any communication method or strategy to inform parents and/or the child that routine weight screening results identified underweight, overweight or obesity. In the context of primary healthcare centres, this is likely to be some form of oral communication, but can involve different educational or counselling strategies. In the context of school health programs, information about weight screening results is likely to be sent to parents as letters or through digital platforms. Combinations of different modes and strategies of delivery are also possible and relevant.	Communicating to parents and/or children about children’s weight status (underweight, overweight or obese) using face-to-face, digital or written interventions or a mix of the above. The intervention must be delivered by a health professional.
**Control:**	1) Usual care	
	2) Other communication method/strategy	
**Outcome**:	Relevant outcomes included, but was not limited to:	
	• Compliance with subsequent activities/referrals	
	• Correct identification of child weight status	
	• Parents’ or the children’s perceptions of the communication with the health care provider	
	• Knowledge and attitudes regarding weight-related issues	
	• Self-efficacy	
	• Experienced stigma	
	• Child’s subsequent weight status	
	• Adverse events/outcomes (any outcome)	
**Language**:	Languages mastered by at least one member of the review team due to the difficulty and time consuming nature of translating qualitative studies (English, French and Scandinavian languages)	
**Year:**	From 2000 to October 2018^a^	

^a^ A cut-off search year of 2000 was used because the millennium development goals were launched in 2000. These goals increased the awareness of the childhood obesity epidemic [31] and this focus was re-enforced by the sustainable development goals [32]

### Search strategy

We developed one comprehensive literature search strategy, covering both research objectives. It was peer-reviewed by a second search specialist and executed in October 2018. We searched nine databases (MEDLINE, PsycINFO, EMBASE, CINAHL, Web of Science, Cochrane Database of Systematic Reviews, DARE, CENTRAL, HTA). The search strategies are available in Additional file 1. The search strategy was developed using guidelines from the Cochrane Qualitative Research Methods Group for searching for qualitative evidence [33] and those for effect review searches [34]. We also searched the reference lists of all the included studies and relevant reviews.

### Study selection

Two researchers independently assessed the publications according to the inclusion criteria, first the title and abstracts, then relevant publications in full text. Disagreements were resolved through discussion or, if required, by seeking a third researcher’s opinion. Where necessary, we contacted the study authors for further information. We note that although language was an exclusion criterion for objective two, we found only publications in a language mastered by members of the review team, thus no records were excluded based on language.

### Methodological quality assessment

All methodological quality assessments were done by two researchers, independently of each other. Any disagreements between the two assessors were resolved by discussion or consensus with a third researcher. For randomised control trials (RCTs), we assessed the risk of bias of each included study using the Cochrane ‘Risk of bias’ tool [34]. For the other study designs, we used study appropriate risk of bias domains as developed by the EPOC group [35]. To assess the methodological quality of included qualitative studies, we applied an adaptation of the Critical Appraisal Skills Programme (CASP) quality assessment tool for qualitative studies. Other reviews of qualitative evidence have also used this tool [36–38].

### Data extraction

We used a data extraction form designed specifically for this review, which included; author, year of publication, geographic setting, description of context, data collection methods (sampling, collection, analysis), description of participants, if ethics approval was given for the study, and results. One researcher extracted data and another checked the completeness and accuracy of the data.

### Synthesis

We sorted the included effect studies according to categories of interventions and control conditions, and assessed results separately for each comparison. We based judgments about whether meta-analyses were appropriate on recommendations in the Cochrane Handbook for Systematic Reviews of Interventions [39]. None of the included effect studies were sufficiently similar to permit statistical pooling of outcome data. With regard to the study by Prina and colleagues [40], we had to transform the numbers for one outcome (attended parent’s information meeting). A statistician imputed the confidence intervals from the reported effect estimates and their associated standard errors using z-statistics. Where possible, two-sided *p*-values were calculated in the same way and compared to the reported p-values.

With regard to the qualitative studies, we conducted a best-fit framework synthesis [41]. Four researchers discussed various frameworks that fit the initial themes identified during data extraction. Through consensus, we decided to use the overarching framework developed in Ames and colleagues [36] about vaccination communication. This framework includes six sections: timing of information; availability of information; amount of information; source of information; content of information; and influence of the relationship between information, the way it is communicated and decisions. In addition to this overarching framework [36], we also decided to use the health belief model [42] to analyse the data about behaviour change related to the influence of the relationship between information, the way it is communicated and decisions. We conducted a thematic analysis [43] within each of the framework areas. During the analysis process, we looked to see if different themes emerged from different participant groups or settings, for example, children, adolescents and parents.

As a final analysis step, we brought together the findings of effect and the qualitative findings. We placed all of the findings into the framework identified for the best-fit framework synthesis to explore differences between the topics investigate by the effect and qualitative studies.

### Appraisal of certainty of the evidence

We assessed our certainty in the findings using GRADE (controlled studies) [44] and GRADE CERQual (qualitative studies) [45].

## Results

### General results

**Fig. 1:**
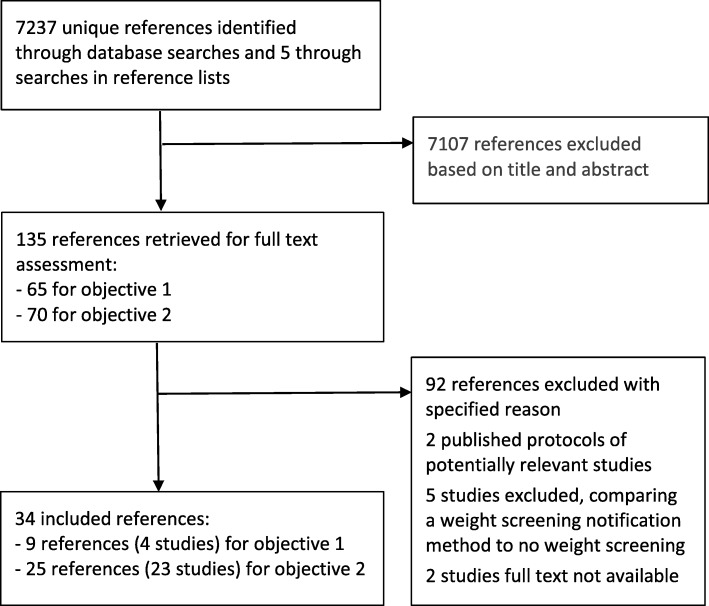
Flow chart for search results and handling of references

The database searches retrieved 7237 references and the manual searches an additional five unique references. We only identified studies reporting on communication and information to children identified as being overweight or obese. None of the included studies reported results related to children identified as underweight. Fig. 1 illustrates the handling of the references. Additional file 2 shows publications read in full but excluded.

### Quantitative synthesis (effect)

We included four effect studies [28, 40, 46–52]. The studies were published between 2014 and 2017, all but one was an RCT, and they were conducted in Mexico, New Zealand, the UK and the US, with a total of 2649 participants (Table 2). All examined different ways of providing weight-screening feedback to parents: face-to-face, various written formats, with or without additional information. Two studies had the same comparison, thus we could group the studies into three comparison groups. We describe the results of these below. Evidence profiles for all the findings can be found in Additional file 4.

**Table 2:** Summary of the characteristics of the included studies of effect

Study ID	Population	Intervention/mode of communication	Comparison/ mode of communication	Outcomes
**Dawson 2014** [28, 46, 48, 49]	New Zealand Health services Families with children aged 4–8.9 years with a BMI above the 85th percentile	Traffic light weight chart combined with motivational interviewing/ Face-to-face interactions with health care providers	Traffic light weight chart with standard conversation/ Face-to-face interactions with health care providers	-Willingness to participate in further treatment of the child -Parental recognition of child’s overweight or obesity -Parental perception of the feedback session -Parental motivation for lifestyle change -Adverse outcomes of the intervention
**Bailey-Davis 2017** [50]	USA Schools Parents with children attending first, third and fifth grade	State-standardised weight-screening report card and easy-to-read information sheet with link to an online screening tool on child’s risk of becoming obese/Written notification	State-standardised weight-screening report card/Written notification	-Parents attended follow up session/ contacted health care provider -Parental perception of the information/ resources given
**Falconer 2014** [51, 52]	UK Schools Parents with children undergoing school-based weight screening	(1) Written feedback and parents of the children identified as obese in two districts received a phone call from a school nurse. (2) Parents in one of these districts were also offered a face-to-face appointment with a school nurse. /Written notification and face-to-face interactions with health care providers	Written feedback with the child’s BMI centile and weight category/ Written notification	-Parental recognition of child’s overweight or obesity
**Prina 2014** [40]	Mexico Schools Parents with children attending second through sixth grade	(1) Written feedback as for the control group and information about the health risks of their child’s weight category. /Written notification (2) Written feedback as for the control group and information about the number of children in the child’s class within each of the weight categories /Written notification	Control: Written feedback with the child’s BMI centile, their weight category and contact information to a nutritionist that could be consulted free of charge. /Written notification	-Parents attended follow up session/contacted health care provider -Parental recognition of child’s overweight or obesity -Child’s subsequent weight status

### Comparison 1: effect of two different formats of face-to-face feedback

A two-phase RCT, conducted in New Zealand, compared the effect of two different formats of face-to-face feedback on a child’s weight-screening results [28, 46–49]. (We do not present results of the second phase, because it describes a treatment program for overweight or obese children.) The study conducted anthropometric measurements of 1093 children, of which the 271 children with BMI > 85th percentile and their families were further assessed. One group received weight feedback using a “traffic light” approach, considered best practice care, and another group received weight feedback using a “traffic light” approach combined with motivational interviewing (MI). Table 3 presents the findings from this comparison and the summary of findings table is available in Additional file 3. The results show that for parents, there is probably little or no difference between the two conditions, with regard to their: attendance of further treatment sessions; recognition of their child’s overweight or obesity; reaction (being upset) about the way information is given; motivation for lifestyle change. However, the parents in the MI condition probably have somewhat greater satisfaction with the way the healthcare provider supports them.

**Table 3:** Table of effect findings comparing feedback using motivational interviewing and feedback using the “stop light” approach

Review finding	Confidence in the evidence	Explanation of confidence in the evidence	Contributing studies
**Source of information**			
**E1:** Parents receiving feedback with motivational interviewing had somewhat greater satisfaction with the way healthcare providers supported them compared to parents receiving feedback using the “traffic light” model.	Moderate	Downgraded by 1 level because of imprecision	Dawson 2014 [28, 46, 48, 49]
**Content of information**			
**E2:** Parents receiving feedback with motivational interviewing had little or no difference in their emotional reaction (being upset) to the way information was communicated compared to parents receiving feedback using the “traffic light” model.	Moderate	Downgraded by 1 level because of imprecision	Dawson 2014 [28, 46, 48, 49]
**Susceptibility of being overweight**			
**E3:** Parents receiving feedback with motivational interviewing had little or no difference in recognizing that their child was overweight or obese compared to parents receiving feedback using the “traffic light” model.	Moderate	Downgraded by 1 level because of imprecision	Dawson 2014 [28, 46, 48, 49]
**Cues to action**			
**E4:** Parents receiving feedback with motivational interviewing had little or no difference in attending further treatment sessions compared to parents receiving feedback using the “traffic light” model.	Moderate	Downgraded by 1 level because of imprecision	Dawson 2014 [28, 46, 48, 49]
**E5:** Parents receiving feedback with motivational interviewing had little or no difference in their motivation to change their lifestyle compared to parents receiving feedback using the “traffic light” model.	Moderate	Downgraded by 1 level because of imprecision	Dawson 2014 [28, 46, 48, 49]

*E stands for effect finding

### Comparison 2: effect of written feedback with or without additional resources

Two studies examined the effect of parents receiving written weight-screening feedback plus additional resources or information in comparison to only receiving written feedback [40, 50–52]. The study designs were RCT and control before and after study (CBA) (embedded in a cohort study), and they were conducted in the USA and UK, respectively. In the RCT, the additional resources were access to web-based information, personal screening, and educational tools. In the CBA study, the additional resources were a call from a school nurse and, in a subsample, a face-to-face appointment. Table 4 gives the findings from this comparison and the summary of findings table is available in Additional file 3. For parents, the results show that there is probably little or no difference between the two conditions, with regard to whether they perceive they get information/resources that help them understand their child’s weight status or help to reduce the risk of overweight, and whether they contact a healthcare provider or perceiving they get useful weight status information. There is insufficient evidence to conclude whether feedback letters plus additional resources, compared to standard feedback letters, improve parents’ ability to classify their child’s weight status or recognise the risks of obesity.

**Table 4:** Table of effect findings comparing feedback letters plus additional resources

Review finding	Confidence in the evidence	Explanation of confidence in the evidence	Contributing studies
**Source of information**			
**E6:** Parents receiving feedback letters plus additional resources had little or no difference in the way they perceive receiving help to reduce their child’s risk of overweight compared to parents receiving a standard feedback letter.	Moderate	Downgraded by 1 level due to unclear risk of bias	Bailey-Davies 2017 [50]
**Content of information**			
**E7:** Parents receiving feedback letters plus additional resources had little or no difference in the way they perceive receiving the information/resources that help them understand their child’s weight status compared to parents receiving a standard feedback letter.	Moderate	Downgraded by 1 level due to unclear risk of bias	Bailey-Davies 2017 [50]
**E8:** Parents receiving feedback letters plus additional resources had little or no difference in their perception that they are receiving useful weight status information compared to parents receiving a standard feedback letter.	Low	Downgraded by 2 levels because of unclear risk of bias and imprecision.	Bailey-Davies 2017 [50]
**Susceptibility of being overweight**			
**E9:** It is uncertain whether parents receiving feedback letters plus additional resources improved parent’s ability to classify their child’s weight status compared to parents receiving a standard feedback letter.	Very low	Downgraded by 3 levels due to study design, risk of bias and imprecision	Falconer 2014 [51, 52]
**Perceived severity of being overweight**			
**E10:** It is uncertain whether parents receiving feedback letters plus additional resources improved parent’s ability to recognise the risks of obesity compared to parents receiving a standard feedback letter.	Very low	Downgraded by 3 levels due to study design, risk of bias and imprecision	Falconer 2014 [51, 52]
**Cues to action**			
**E11:** It is uncertain whether parents receiving feedback letters plus additional resources contacted a healthcare provider compared to parents receiving a standard feedback letter.	Low	Downgraded by 2 levels because of unclear risk of bias and imprecision.	Bailey-Davies 2017 [50]

*E stands for effect finding

### Comparison 3: effect of three different formats of written feedback

Lastly, we included an RCT from Mexico that examined the effect of three different formats of written feedback letters to parents after school-based weight screening (results of a fourth group receiving no information is not included in this review) [40]. The letters differed with regard to whether BMI and health information was presented i) without comments, ii) with messages about the health risks, or iii) with information about other children’s weight status. The parents of 824 children identified as obese and overweight receiving any of the written weight-screening feedback letters are included in the analyses. Table 5 presents the findings from this comparison and the summary of findings table is available in Additional file 3 The results show that for parents, there is probably little or no difference between the three feedback conditions, with regard to whether they attend parents’ information meetings and in taking any action to address their child’s BMI. Similarly, there may be little or no difference between the three feedback conditions with respect to the children’s subsequent BMI, but parents may have somewhat lower ability to classify their child’s weight status correctly when they only receive simple written feedback.

**Table 5:** Effect findings comparing different formats (phrasing) of written weight screening feedback letters

Review finding	Confidence in the evidence	Explanation of confidence in the evidence	Contributing studies
**Susceptibility of being overweight**			
**E12:** Parents receiving different formats (phrasing) of written weigh-screening feedback letters may have somewhat lower ability to classify their child’s weight status correctly when they receive simple written feedback.	Low	Downgraded by 2 levels because of unclear to high risk of bias and imprecision	Prina 2014 [40]
**Cues to action**			
**E13:** Parents receiving different formats (phrasing) of written weigh-screening feedback letters have little or no difference in taking action on their child’s weight.	Moderate	Downgraded by 1 level because of unclear to high risk of bias	Prina 2014 [40]
**E14:** Parents receiving different formats (phrasing) of written weigh-screening feedback letters have little or no difference on their child’s subsequent weight status.	Moderate	Downgraded by 1 level because of unclear to high risk of bias	Prina 2014 [40]
**E15:** Parents receiving different formats (phrasing) of written weigh-screening feedback letters may have little or no difference in whether they attend a parent’s information meeting.	Low	Downgraded by 2 levels because of unclear to high risk of bias and imprecision	Prina 2014 [40]

*E stands for effect finding

### Qualitative synthesis

We included 23 qualitative studies, presented in 25 publications [53–77]. As summarized in Table 6, 15 studies were conducted in the USA [53, 54, 56, 57, 59–63, 65, 68–70, 72, 74–76], five in the United Kingdom [55, 58, 66, 67, 77], and one each in Australia [71], Canada [64] and Norway [73]. Twelve of the studies concerned information sent from elementary/middle schools or preschools [53, 55, 58, 60, 61, 66–70, 72, 76, 77], 11 regarded face-to-face communication with healthcare providers in primary healthcare centres [54, 56, 57, 59, 62, 64, 65, 71, 73–75], and one study explored parental preferences regarding communication about their child’s weight [63]. Parents were participants in 21 studies [53, 54, 56–65, 67–77], 10-year-old children the participants in two studies [55, 66] and children/adolescents in three studies [57, 64, 71].

**Table 6:** Summary of the characteristics of the included studies of experience and expectations (qualitative studies)

Study ID	Country	Participants	Mode of communication and setting
Alba 2018 [53]	USA	Parents of overweight and obese elementary school students in south eastern Pennsylvania where one third of the population is economically disadvantaged	Letter sent home from elementary school
Ayash 2012 [54]	USA	Parents of children with a BMI above the 85th aged 2 to 13 years in Massachusetts where low-income, young, black and Latino children are most effected	Face-to-face interactions with exploration of preferences regarding receiving a letter before or after the appointment
Blood 2011 [55]	United Kingdom	Children aged 10–11 who had gone through weight screening in the last two months	Face-to-face weight screening experience
Bolling 2009 [56]	USA	Mostly white, privately insured suburban, urban and rural parents of children aged 2 to 6 years and between the 85th and 94th percentile body mass index in the suburban mid-west.	Parental preferences for terminology related to weight at health visits
Bossick 2017 [57]	USA	Teen patients from metropolitan Detroit diagnosed as overweight in the last 12 months and mothers	Face-to-face meetings with health care providers
Gainsbury 2018 [58]	United Kingdom	Parents of 4–5 year olds in south-west England who had recently received written feedback from the national child measurement program representing the full spectrum of feedback options (under-, healthy, over- and very overweight)	Letter from school setting
Gillison 2014 [77]	United Kingdom	All parents receiving letters informing them that their child was overweight (91st –98th centile) or very overweight (98th–100th centile) in south-west England	Letter from school setting
Guerrero 2011 [59]	USA	Low-income Spanish speaking Mexican mothers of children ages 2–5 years attending a free clinic	Face-to-face meetings with health care providers
Harris 2009 [60]	USA	Students and parents from an area in West Virginia with high levels of obesity, underserved by medical professionals, rural and with low socio-economic status	Letter from school setting
Jorda 2017 [61]	USA	Parents in Florida who had received BMI referrals for their children in first, third or sixth grade and child was over the 95%. The obesity rate for the area was 40%.	Letter from school setting
Knierim 2015 [62]	USA	Self-identified Latino, 18 to 80 years old, and the parent or grandparent/primary caregiver of a 2- to 18-year-old primary care patient in a poor area of Denver, Colorado with a high prevalence of obesity in the Latino community	Face-to-face meetings with health care providers
Kubik 2007 [63]	USA	Parents of elementary school students from a suburban school district in Minnesota	Exploring how parents wanted to receive communication about their child’s weight
McPherson 2018 [64]	Canada	7–18-year olds with and without disabilities and their caregivers from two large paediatric hospitals in Ontario	Face-to-face meetings with health care providers
Moyer 2014 [65]	USA	Parents/caregivers of 8- to 14-year-old obese (95th BMI-for-age percentile) children from low income families attending public schools in Massachusetts	Letter from school setting and face-to-face meetings with health care providers
Nnyanzi 2016 [66]	England	Children who had been weighed at school aged 10–11 in the North East of England in an area with a relatively high prevalence of childhood obesity	Letter home to parents from school setting as well as the experience of being weighed at school
Nnyanzi 2016a [67]	England	Parents/guardians after they had received their child’s weight results letter in the North East of England in an area with a relatively high prevalence of childhood obesity	Letter home from school setting
Ruggieri 2013/2016 [68, 76]	USA	Parents of children in grades Kindergarten- grade 8 in a school district in Philadelphia. Had to be English speaking so excluded Latino families with a higher prevalence of obesity.	Letter home from school setting
Schwartz 2010/2015 [69, 70]	USA	Parents of children who had received a letter stating their child was overweight in the Mid-West	Letter home from school setting
Shrewsbury 2010 [71]	Australia	Adolescents and unrelated parents of adolescents from low-middle socio-economic areas in Sydney and the surrounding area	Face-to-face communication with a health care provider
Thompson 2015 [72]	USA	Parents who identified as Latino, non-Hispanic white, African American, or Asian American in a low income area	Letter home from school setting
Toftemo 2013 [73]	Norway	Parents of overweight children aged 2.5–5.5 years in a rural part of eastern Norway	Face-to-face communication with a health care provider
Valencia 2016 [74]	USA	Mostly Latino mothers and caregivers attending clinics for low income families in southern Arizona	Face-to-face communication with a health care provider about growth charts
Woolford 2007 [75]	USA	Mothers of pre-schoolers recruited from a Head Start program for low income families in Michigan	Face-to-face communication with a health care provider

In the following section we present a summary of the qualitative findings identified during the best fit framework synthesis. The categories from the framework are used to group the summaries. For the individual findings within each framework category and our confidence assessments see Table 7.

**Table 7:** Summary of qualitative findings

Review finding	Confidence in the evidence	Explanation of confidence in the evidence	Contributing studies
**Timing of information**			
**Q1**: Some parents felt that there was a lack of communication about the weighing and notification process. They wanted information about the weighing process before the testing occurred to know what to expect and again before the results were sent home in order to be prepared to receive the letter. They wanted the information to be up to date with recent measurements.	Moderate	Minor concerns: methodological limitations Major concerns: relevance	[53, 54, 61, 70, 76]
**Availability of information**			
**Q2:** Many parents believed that they should be asked to give consent for weight screening and the option to opt out. They felt that they had not received this information. Due to this, they felt that they had not had the option to give consent or opt out.	Low	Minor concerns: adequacy Moderate concerns: methodological limitations Major concerns: relevance	[60, 61, 76]
**Q3:** Many parents disliked that the information about and permission for testing was sent with other school documents which led to it being lost, not seen or not remembered. Parents wanted follow up information about nutrition and health sent separately from the results letter for the same reason.	Low	Moderate concerns: relevance Major concerns: adequacy	[53, 61, 67]
**Q4:** A few parents were frustrated that the school did not provide a platform for parents to give feedback on the weighing process and communication/notifications about it.	Very low	Major concerns: relevance and adequacy	[53, 67]
**Q5:** Parents had varied opinions about whether all children should receive weight notification or only those children who fall outside of the healthy range. Parents who believed all children should receive notification were concerned about privacy and confidentiality. Those who believed only those who fall outside of the healthy weight should receive notification were concerned about the cost of sending notifications.	Low	Major concerns: relevance and adequacy	[63, 69, 70]
**Amount of information**			
**Q6**: Many parents wanted more information about how to interpret the screening results they received in letters and growth charts. Many felt that they had limited knowledge and understanding of how to interpret the results and needed further explanation and assistance.	Moderate	Minor concerns: methodological limitations Moderate concerns: relevance	[53, 54, 65, 70, 73–77]
**Q7:** Many children wanted more information about the weighing process before, during and after the process itself. For example, and introduction session and a follow up session. This lack of information can make them feel nervous, terrified or unsure.	Moderate	Minor concerns: coherence and adequacy Moderate concerns: relevance	[55, 66, 71]
**Source of information**			
**Q8:** Health care providers were a trusted source of information about a child’s weight and could influence parental motivation to address a child’s weight issues. Parents and adolescents felt weight assessments done by health workers were useful, took their advice seriously, and expected that it was their role to inform them about weight issues. They wanted the clinician to approach the weight conversation first in a sensitive, respectful, direct and positive manner using open questions. They wanted health care providers to be proactive in raising the topic, be forthright in their discussions, provide clear messages and in some cases link the child’s excess weight to health risks. They wanted the provider involved in developing a follow-up plan and to share the responsibility for the plan. Some preferred the HCP and did not want the school involved.	Moderate	Minor concerns: methodological limitations and relevance	[53, 54, 56, 57, 59–65, 70, 71, 73, 74]
**Q9:** Parents wanted HCPs to intervene early and initiate conversations if they were concerned about a child’s weight and customize or tailor the weighing and communication process to each child.	Moderate	Minor concerns: adequacy Moderate concerns: relevance	[54, 56, 57, 64, 73, 74]
**Q10:** Parents felt that there were long wait times to see their HCP and when they were seen that appointments were rushed.	Very low	Minor concerns: coherence Moderate concerns: methodological limitations Major concerns: relevance and adequacy	[57, 74]
**Q11:** The way that HCPs reacted to the weight screening letter from the school or discussed the child’s weight led parents to believe or dismiss the screening results.	Low	Minor concerns: methodological limitations and adequacy Major concerns: relevance	[53, 69, 70]
**Q12:** Many parents approved of receiving a letter delivered by confidential standard mail to communicate screening results. Many did not approve of sending the letter home with the child. Those who did not approve of the letter wanted a more personal form of communication such as a phone call, email or face-to-face meeting.	Moderate	Minor concerns: methodological limitations Major concerns: relevance	[53, 54, 60, 61, 63, 65, 68, 76]
**Q13:** Secrecy, privacy and confidentiality were important to both children and parents during (conducted in a private and confidential manner) and after (who has access to the results and how they are delivered to parents) the weighing process. Participants were concerned with privacy in order to avoid teasing, bullying, embarrassment and stigma and in some case parents wanting to control access to the screening results so that children could not see them. However, some children wanted the social support of their friends while being weighed and measured.	Moderate	Minor concerns: methodological limitations Major concerns: relevance	[53, 55, 60, 61, 63, 65, 68–70, 76]
**Q14:** Many parents wanted more individual follow up and specific, concrete, practical and age appropriate support and guidance for lifestyle changes for instance through additional information, guidance, supplemental materials or referrals to relevant programs. When this was not done, or felt to be lacking, it led to frustration and confusion and was often experienced as a barrier to addressing their child’s weight issue.	Low	Minor concerns: coherence Moderate concerns: methodological limitations Major concerns: relevance	[53, 54, 57, 60, 63, 67, 69, 70, 72]
**Content of information**			
**Q15:** Parents had clear preferences for the format, content, presentation, literacy level and tone of the weight notification letters they received. Many felt that the letter lacked necessary information or wanted more information included to help them take to steps to improve their family’s health. Importantly, they wanted a simple, easy to understand, visual explanation of BMI and how to interpret the results.	Moderate	Minor concerns: methodological limitations Major concerns: relevance	[53, 54, 60, 63, 67–70, 72, 76, 77]
**Q16:** Parents had clear preferences for terminology used in letters and health care providers when discussing/presenting the issue of children’s weight. This choice of terminology could communicate respect and promote engagement. These clear preferences for the terminology being used included specific words, to avoid judging, insulting or the feeling that parent’s worries were not being taken seriously. If parents felt defensive, judged or offended they sometimes refused to return to the provider.	Moderate	Minor concerns: methodological limitations Major concerns: relevance	[54, 56, 61, 62, 64, 65, 72, 75]
**Q17:** Language barriers and not having translators limited communication between parents and the health services. When language barriers arose, parents were often given written materials instead of discussing the child’s situation with the provider. This limited communication was a barrier to growth monitoring.	Very low	Moderate concerns: methodological limitations Major concerns: relevance and adequacy	[54]
**Perceived susceptibility of being overweight**			
**Q18:** Some parents expected and accepted the results of the BMI letter and were not surprised. However, the majority of parents did not accept the results of the BMI letter. They did not consider their child overweight. They questioned the credibility of the process, the accuracy of BMI measurements, and that the letter varied from the information given by their health care provider. The feedback they were given did not match their perception of their child and the weight report was often discounted. Many viewed the letter as a judgement or criticism of their parenting.	Moderate	Minor concerns: methodological limitations Moderate concerns: regarding relevance	[53, 58, 60, 61, 65, 67, 70, 73, 77]
**Q19:** Children who were overweight often were surprised by the results and entered a phase of denial or shock. They also question if the measurements were right as they felt the results must be a mistake. Weight results could cause changes in social structure among children as they start to identify with others who are the same as them. Many children reacted emotionally to learning their weight status. Those who were overweight often reacted with negative emotions or disbelief, which influenced their mental health and well-being and caused worry. Children who were normal weight often reacted with joy and happiness at the results.	Very low	Minor concerns: adequacy Major concerns: relevance	[66, 70]
**Q20:** Many parents participated in an ‘othering’ process when receiving feedback about their child’s weight. This process contributed to the dismissal of overweight feedback received by themselves or their non-othered peers using language to define themselves and separate them from the ‘other’ parents whom they perceived needed to be the target of obesity prevention and that these ‘others’ were often not listening. Another group, parents of normal weight children, believed that they were part of the group doing the right thing and viewed other people, especially those whose children were indicated to have weight problems as not doing things correctly.	Moderate	Moderate concerns: relevance	[58, 61, 67]
**Perceived barriers to addressing weight issues in the school system**			
**Q21:** Parents commented that on one hand the school was doing the BMI measuring but on the other hand, in most cases, was not making changes to facilitate activity and healthier lifestyles for students within the school environment.	Very low	Minor concerns: coherence Moderate concerns: adequacy Major concerns: relevance	[53, 61, 68, 70]
**Cues to action**			
**Q22:** Many parents had an emotional response to being informed about their child’s weight, who was informing them about their child’s weight and their child’s weight. These varied from positive/neutral, negative, disbelief and more than one emotion. Often parents cycled through the emotions. This reaction was often tied to the child’s weight status with those receiving healthy weight notifications being most positive. A parent’s emotional reaction could influence their perception of the screening program and the school and their motivation to act.	Moderate	Minor concerns: methodological limitations Moderate concerns: relevance	[53, 58, 60, 61, 65, 67, 69, 70, 77]
**Q23:** In some cases, parents said that receiving the letter about their child’s weight had been a cue to action. Other parents ignored, downplayed or dismissed the letters and took no action and for some their level of concern did not change. A few parents said the letter had no impact as they had already implemented changes in their household before receiving it and continued with these.	Moderate	Minor concerns: methodological limitations Moderate concerns: relevance	[53, 61, 67, 69, 70, 77]
**Self-efficacy**			
**Q24:** Many parents discussed their struggles with self-efficacy and their ability to make changes at home. Some felt concerned, hopeless and overwhelmed when it came to choosing which changes to make and how to implement them. They mentioned a lack of knowledge, access to services and finances.	Low	Minor concerns: methodological limitations Moderate concerns: relevance and adequacy	[54, 69, 70, 73]
**Q25:** Many parents felt they lacked knowledge about how to communicate to their children about their weight or changing habits. They found this distressing and it caused fear and frustration. Some parents did not want children to see the letter or hear the results of their screening for fear of causing harm to self-esteem or body image. Other parents still chose to discuss the screening results with their children but feared doing harm. Many parents felt that involving a child in these discussions should be tailored to the child’s age. Parents wanted guidance and kid friendly suggestions for communicating to children about their weight.	High	Minor concerns: methodological limitations and coherence	[53, 57, 60, 64, 67, 70, 71, 73, 77]
**Q26:** Some children felt that they had limited information about what they can do about their weight situation. They rely on parents and guardians for information about what can be done.	Very low	Minor concerns: methodological limitations Major concerns: relevance and adequacy	[66]

*Q Stands for Qualitative finding

### Timing of information

Some parents felt that there was a general lack of communication about the routine weight screening- and notification process [53, 70] and that the notification process prior to weighing was weak [53, 61, 70, 76]. Others wanted to be notified about when to expect the weight screening results in the mail so that they could prepare [53, 61] and that the information should be sent out quickly so that it is up to date with recent measurements [54].

### Availability of information

Although schools provided a letter at the beginning of the school year to opt out of the weight screening, many parents did not remember receiving or seeing this letter [61]. Some parents felt that the screening had taken place without their knowledge, “behind their back”, when the referral letter arrived home without warning [61, 68]. This issue also applied to the follow up information received by parents that often accompanied the weight notification letter. Many parents confessed that the supporting information they received with the letter was not seen, disregarded or placed in the bin often due to the emotional reaction to the letter itself. Some suggested that it would be better to send this supporting information later once the parent had absorbed the results from the notification letter [67].

### Amount of information

Many parents were aware of growth charts and BMI weight reports and felt that they were useful tools [73]. However, they were unsure of how to read and interpret them [65, 74], and needed and wanted a better explanation to understand them [53, 73, 76, 77]. When there were no explanations, parents often misunderstood the growth charts and BMI weight reports [70, 75].

Many children found the weighing process to be secretive [55]. They did not know what to expect [55] and this could cause fear and anxiety [55, 66]. Children who were familiar with being weighed at home did not experience the same fear or worry [66]. To make children feel more comfortable authors recommended an introduction session before weighing and a drop in session after to discuss questions and concerns the children had [55].

### Source of information

Most parents agreed that healthcare providers played an important role in addressing their child’s weight [54, 56, 57, 59, 71, 74] and reported high trust in providers [57, 64, 74]. This trust could lead to greater comfort with the provider and feelings of better quality of care [57, 64]. Some parents felt that it was not the role of the school system to comment on their child’s weight [53, 65, 70]. They felt more comfortable and preferred to have their healthcare provider address weight issues [53, 59, 60].

Parents had clear expectations of the healthcare providers. They should be forthright, direct, address and initiate conversations about weight [54, 56, 57, 59, 73], thus taking the pressure off parents to initiate a difficult discussion [54]. They should use a sensitive approach [54, 57, 71], be positive [56, 57, 65], show interest [56], intervene early [56, 73], and talk directly to the child in a caring positive manner [54, 57, 65], sending a clear message [56]. Parents and children also had a preference for the use of open ended questions in a respectful tone [64] and motivated by concern for the child [65]. They wanted them to present and discuss the health risks associated with being overweight [56, 62, 65]. They also wanted support from their healthcare providers in developing a step-by-step specific, practical and individualised plan and accessing local information to support behaviour change [54, 57, 61] and to explain these concepts in a way that both parents and children could understand [65]. Some parents and children also felt that healthcare providers should tailor the conversation to the child’s age and be flexible about when children should be involved in the conversation and how often the conversation should take place [64].

Adolescents (aged 14–16) and parents felt that the adolescents took information coming from providers more seriously and responded better to them [57]. Adolescents also reflected on the providers’ intentions, reporting them as being supportive and interested and that this motivated them to change health behaviours [57].

Confidentiality and privacy were important during the screening process. Parents and children felt strongly that weight screening should be performed in a private setting [55, 60, 61, 63, 69, 70, 76] in order to avoid embarrassment, teasing and stigmatisation [60, 61, 63, 65, 76].

Some parents experienced that the weight screening results from their healthcare provider were different from those received from the school, or the healthcare provider’s reaction to the school screening led them to question or totally disregard the school results [53, 70].

The majority of parents who talked about their experiences with and preferences for information approved of the information being sent home by letter, but had concerns about how the letter would be sent [53, 61]. Confidentiality and privacy were important with regard to the delivery of the weight screening results [61, 68–70]. They preferred delivery by standard mail directly to them [53, 60, 65, 76]. Some parents did not want the letter sent home with the child [60, 61, 63, 76], as they were concerned that if the letter was given to the child, the child could open, forget or discard it [53, 61, 63, 76]. Parents did not like that the child might see the letter first, as they worried that this could have a negative impact on the child [65]. Parents who did not approved of the weight screening information being sent home by letter wanted a more personal form of communication, for example, a call from the school nurse [53, 61], having teachers deliver the information at parent-teacher conferences [63] or email [53, 54].

Some parents wanted additional materials for addressing a child’s above normal BMI and for the family in general, such as websites, phone numbers, information letters or pamphlets [53, 54, 57, 63, 70, 72]. These parents felt that a letter with an explanation of the weight result was not enough to support them with further action and decision-making [53, 67, 70, 72]. Some mentioned that they also lacked support, such as a support hotline to phone, after receiving the weight notifications [53] and wanted links to local programs or resources [54]. When parents experienced that follow up and guidance were lacking, some experienced frustration and confusion [54, 57, 67]. This was also seen as a barrier to addressing the child’s weight issue [53, 54, 57].

### Content of information

Some parents expressed concerns about the content of the weight screening notification letter [53]. One of these concerns was the verbiage [53] and the format of the letter [53]. Parents wanted a simple, easy to understand, visual explanation of BMI and how to interpret the results [63, 72, 77]. Some felt that the letter was too general [53, 70], impersonal [53], and many parents felt that the content of the letter lacked necessary information [53, 63, 76, 77] (See Table 8).

**Table 8:** Information parents felt was lacking from the information letter

• A better explanation regarding it’s purpose [53, 76]	
• A clear statement of findings [60, 72]	
• The procedures used and timeframe for when measurements took place [53, 76]	
• Additional materials for addressing above normal BMI [53, 60, 63, 70, 72]	
• Health risks to help parents recognize the potential long term consequences of a child being overweight or obese [69]	
• A better explanation of how to interpret BMI data [63, 76, 77]	
• Provision of more individually tailored information [69, 70, 77]	
• How the results will be kept confidential [76]	
• How the BMI screening program fits within the school districts’ larger plan to address overweight and obesity [76]	
• Pictures and visual representations such as stoplight colours to represent BMI [72]	

Some parents felt that the tone of the weight screening notification letter was judgemental and negative [53]; judging their parenting abilities [53, 67] or insulting their child when words like overweight were put in bold [67]. Parents wanted the letter to be written using sensitive language and a supportive tone [54]. Parents suggested that the content of the letter should focus on what the whole family can do, rather than just the target child [63, 72].

Parents stressed the importance of appropriate terminology in order to communicate respect and promote engagement [64]. They believed that healthcare providers should explore a family’s preferred terminology when communicating about a child’s weight [64]. In many cases, parents preferred the terms overweight and obese [56, 64] and suggested to use these in reference to national norms to aid in understanding [56] or to discuss health and growth rather than weight and size [64]. Still, some parents avoided using the term obese altogether as they found it to be an untrue description of their child, hurtful, insulting and judgemental [61, 62, 65, 72]. They considered colloquial terms such as fat, chubby or plump offensive and inappropriate for use in official letters or interactions with healthcare providers [56, 64].

Some parents said that hearing the word overweight would be motivating and convey a strong message [56]. They felt the same way about the term obese as it would be an “eye opener” [56, 75]. However, terms like “at-risk for overweight” and “unhealthy weight” and “normal weight” were vague and confusing and would not motivate them to take action [56, 62, 72].

The next section presents findings related to the influence between the relationship of information, the way it is communicated and the action taken by participants. The headings in this section represent the sub areas of the health belief model which was the framework used to analyse the data in this section.

### The perceived susceptibility of being overweight

A few parents accepted the results of the weight notification letter [53, 61, 65, 67, 70]. These parents mentioned finding the letter helpful and welcomed it compared to a lack of notification in the past [65]. Others said it confirmed what they already knew [53, 61, 67, 70]. Parents who had overweight children and who accepted the letter, viewed it as an opportunity to make some needed changes [70] and were happy that their child’s weight issue had been brought to their attention [70].

Many parents questioned the results they received from the BMI testing [53, 60, 77]. They described receiving feedback about a child’s overweight in negative terms [58, 61, 65, 67, 69, 70], using words such as “cross”, “angry”, “annoyed”, “upset”, “insulted”, “distressing” and “perturbed” to describe how they felt [58, 61, 67, 70]. The letter caused a great deal of panic and worry among parents of overweight children, as they felt they had been caught unawares [67, 70]. Other parents felt as if they were being judged [58, 77], responding with anger and defensiveness in some cases [77]. Some parents felt disappointment in not being able to live up to their own expectations for themselves in regards to managing their child’s weight [77].

Some parents disregarded, ignored, disagreed with or did not believe the results they received from the weight notification letter [53, 58, 61, 70, 73, 77]. Several study authors believed that this disagreement highlighted the misconceptions that parents have regarding their child’s weight classification [53, 58, 60, 61, 67, 70, 73, 77]. Some parents had received contradictory remarks from the child’s healthcare provider [53, 61, 70]. Parents who did not believe the school’s findings or disregarded the letter had a lower perception of the severity of being overweight and/or a decreased level of concern about the problem [53].

The authors of one study [66] found that children reacted very differently to their weight notification. Children receiving normal weight feedback often reacted with joy and happiness. However, children who were told they were overweight were often surprised about the result, entering a phase of denial or shock. Many felt that the results must be a mistake and questioned if the measurements were right. The reactions to weight feedback were often very emotional, with those who were overweight reacting with negative emotions or disbelief. This caused a lot of worry, which in turn could influence their mental health and well-being.

One group of parents who had overweight children participated in an othering process (to view or treat someone as intrinsically different from you) where they believed their children were fine and not the target group of the BMI measurement program, but others with overweight children were [58, 61, 67]. Parents of normal weight children also participated in othering [58, 67]. This othering process allowed parents/guardians to identify themselves as part of the group that is doing the right thing, and viewed others, especially those with overweight children, as not doing things correctly [67]. The process of othering contributed to the dismissal of overweight feedback that parents received themselves, legitimising their rejection of the feedback for their child [58, 67]. Parents used distinct language to define themselves from the other group who they perceived did need to be targeted [58, 61]. Parents described themselves as; educated, responsible, middle class, and interested [58]. They described the other parents as; irresponsible, ignoring healthy living advice, and fed their children unhealthy foods [58, 67]. Many also believed that these others who did need to change were not listening [58] and so questioned the impact of the notification letters [58].

### Perceived barrier to addressing weight issues in the school system

Some parents felt that the school was not doing enough to address the results of the weight screening as students spent more of their time in school environments than at home [53]. Parents suggested more time for activities such as recess or gym time [53, 68] or offering healthier food options [53, 61, 68].

### Cues to action

Some parents discussed how receiving the weight notification letter had been a cue to action for them [53, 61, 67, 69, 70, 77]. They used the letter as a tool, showing it to their children [53, 70] or spouses, friends or neighbours [67, 70] to start a discussion and create awareness and opportunity [53, 70]. Some implemented changes or planned to implement changes in diet and activities with or without the knowledge of their children [53, 61, 67, 70, 77]. Finally, for some the letter was a cue to action to contact their family physician for follow up [70].

Other parents ignored, downplayed or dismissed the letter, taking no action to address the weight of their child [53, 61, 67, 77]. This was often because they were not fazed by the results so ignored or downplayed the severity of the information they had received [53, 61], stating that their child was already very active and/or was eating a healthy diet and so did not need to implement changes [61, 67]. For others, they just did not believe the results of the letter and were angry at receiving it [61]. Finally, some parents said the letter had no impact as they had already implemented changes in their homes to address weight issues before receiving the letter and so continued with these [53, 67].

### Self-efficacy in addressing children’s weight issues

Many parents talked about how difficult they found it to control their child’s weight [53], expressing feelings of concern, lack of knowledge, hopelessness and being overwhelmed [53, 70]. Many felt that despite trying to make changes in eating and exercise habits they were unable to significantly reduce their child’s weight [53]. They were unsure of where to go for help and what actions to take [70].

Many parents found it difficult to talk to their children about their weight [70, 73]. They found it stressful as the children would often become emotional and shut down and parents were unsure of how to react [70]. Many parents felt that they lacked the knowledge on how to communicate with their children about the topic leading to fear and frustration [70]. They were unsure of how to respond when their children started commenting on their own weight as well in order not to have a negative impact, for example, on self-esteem [70]. Parents wanted to know more about how to discuss BMI findings with their children, including kid friendly suggestions to use in the family [53].

Some parents felt a lack of knowledge and fear of doing harm when discussing weight issues with their children, parents want guidance in how to talk to their children about their weight notification. Some parents preferred to discuss their child’s weight without the child present either between themselves or with a health care provider as they feared the child would understand the conversation and this could lead to the development of low self-esteem or eating disorders [64, 71, 73]. Some parents felt that it was important to consider the child’s age when deciding if they would be involved in the conversation [57, 64, 71] or if the child was older whether the parent would be involved in the conversation [71]. Other parents supported a phased approach where the child would be increasingly included in the conversations over time [64]. Others chose to not have any conversations with their children about the letter as they did not want their child to think they were overweight or be labelled or believed that talking about it could lead to other problems [67, 70].

Some children felt that they had limited information about what they themselves could do about their weight. They had to rely on the adults around them, their parents and guardians, for information about how to tackle their weight issues. This lack of information about what action they could take often caused the children to worry [66].

### Bringing together the effect and qualitative findings

Above, the results of the studies about effect and the studies about experiences are presented separately. Here, we show the results of placing all of the findings into one framework (Table 9). It shows that the effect studies had a narrower scope of research covering five framework areas; source of information, content of information, perceived susceptibility of being overweight, perceived severity of being overweight, and the parent’s cues to action. The research focus and findings from the qualitative studies were broader, covering all but two areas of the framework; the perceived benefits of being overweight and the perceived severity of being overweight.

**Table 9:** Overarching framework with all findings

	Timing of information	Availability of information	Amount of information	Source of information	Content of information	Influence between the relationship of information, the way it is communicated and action (using the health belief model)					
						Susceptibility of being overweight	Perceived severity	Perceived benefits	Barriers to addressing weight issues in schools	Cues to action	Self-efficacy
**Effect findings**				E1^b^	E2^b^	E3^b^	E10^d^			E4^b^	
				E6^b^	E7^b^	E9^d^				E5^b^	
					E8^c^	E12^c^				E11^c^	
										E13^b^	
										E14^b^	
										E15^c^	
**Qualitative findings**	Q1^b^	Q2^c^	Q6^b^	Q8^b^	Q15^b^	Q18^b^			Q21^d^	Q22^b^	Q24^c^
		Q3^c^	Q7^b^	Q9^b^	Q16^b^	Q19^d^				Q23^b^	Q25^a^
		Q4^d^		Q10^d^	Q17^d^	Q20^b^					Q26^d^
		Q5^c^		Q11^c^							
				Q12^b^							
				Q13^b^							
				Q14^c^							

^a^High confidence in the finding

^b^Moderate confidence in the findings

^c^Low confidence in the findings

^d^Very low confidence in the findings

The findings once placed in the framework show that future effect studies could also look at the impact of the timing of the information to parents, information availability, the amount of information parents and children would like to receive, as well as issues related to barriers to addressing weight issues in schools and feelings of self-efficacy.

## Discussion

We identified four reviews that explored a topic of interest close to the one explored in this mixed methods review; communication about children’s weight. In contrast to our review which examined weight notification, Mogul 2014 [78] studied whether family communication strategies used in addiction treatment could be used in paediatric obesity weight management programs. They found that unhealthy communication patterns and parental restrictions were related to maladaptive eating patterns in children and attrition from weight loss programs. However, no studies had concrete suggestions to aid family communication around issues of food and weight.

Mikhailovich 2007 [79] explored childhood obesity and overweight with parents and what is known and what might be helpful for health care providers when discussing a child’s weight with the child and the parents. They identified factors that can influence the discussion about a child’s weight and the child’s weight in general including; demographic, work, time and lifestyle related, parental underestimation of children’s weight, parents’ perception of weight management, peer pressure and pester power, stigma, health care provider attitudes and practice, health care provider knowledge and skill and communicating difficult news. Many are reflected in our findings, especially how parents expect and want health care providers to interact with them and their children, fears of stigmatization and the want for clear and supportive information.

McPherson 2017 [80] aimed to identify and synthesize the available evidence on weight communication. They included the viewpoints of health workers, parents and children and examined communication in health settings. They did not include school health programs. Communication was not limited to informing about the child’s weight status but looked at all weight communication including treatment and follow up. Some of the trends identified are similar to our findings. All participants should be involved in discussions about weight, the topic of weight should be raised early and discussed often, there were clear preferences for the terminology used in discussions and that discussions should be augmented with appropriate tools and resources.

Finally, Davidson 2018 [81] identified and compared school based weight assessment programs containing feedback to parents from OECD countries. They found that the majority of OECD countries do not currently have such programs. Successful programs have high levels of political and social support as well as collaboration among the public health sectors, schools and parents. Similar to our findings, they also comment on the importance of health service providers being accessible and involved in following up when a child is identified as overweight or obese.

We did not identify any studies that reported results related to children identified as underweight.

We also identified relevant reviews that address the findings of this systematic review related to communication on different health topics as well as tailoring of health information. Similar parental preferences for early, clear, tailored and easy to understand information from health professionals were identified in a qualitative evidence synthesis on parental preferences for information about childhood vaccinations [36, 82] and decision support needs of parents making child health decisions [83]. A meta-analysis of tailored print health behaviour change interventions found that tailored interventions were more effective than non-tailored interventions for health promotion [84]. Research on promoting understanding and engagement with digital behaviour change interventions has found that successful intervention design demands a user-centred and iterative approach [85]. This type of research design could be used to develop weight assessment feedback forms in conjunction with parents to address their needs and preferences leading to a potentially higher level of acceptance and engagement with the screening results.

### Strengths and limitations

Our systematic review comes with strengths and limitations. A strength of this mixed methods systematic review is the close collaboration between the commissioner and the research team in coming to an agreement on the objectives, protocol, and types of studies to be included. This ensured high relevance. Further, we used systematic and transparent methods throughout the review process, and combined evidence from both experimental studies about effect and qualitative studies about people’s experiences and perspectives. By viewing such studies through the same lens, it is possible to enhance the/our understanding of how the findings interrelate. With regard to limitations, our literature search is more than a year old, and it is possible that new relevant studies have been published after this date. We relied on the information and data presented in the published articles, which in turn are limited by issues such as word restrictions. Children identified as underweight were not covered in any of the included studies.

As part of the qualitative synthesis process, the authors working with objective 2 reflected on how our backgrounds and positions might have influenced our choice of review topic, study selection, data extraction, analysis, and interpretation of data. Our backgrounds are in health systems research, social sciences, and pedagogy and, while working on the synthesis, we were all employed by The Norwegian Institute of Public Health. None of the reviewers have been involved in primary research related to weight assessment programs or communicating to parents or children about their weight. HA has been involved in research related to childhood vaccination programs where she routinely saw children being measured and weighed but weight and weight feedback were not the focus of the research. Before working on the synthesis, we did not have any preconceived ideas regarding weight assessment and weight status notification interventions. However, we believed that the implementation of programs should be informed by robust evidence of effectiveness, acceptability and feasibility.

### Implications for practice

The following questions, derived from our findings, may be helpful to consider when implementing or planning for routine childhood weight screening communication strategies in order to address issues of importance to their target population. It is important to consider local contextual factors including gender, age, cultural group, and education when implementing new strategies for communicating with parents and children about their weight status. Consider:

1. Is information about weight screening and weight notification communicated to parents and/or children in good time before the process begins and again before the results are sent home to let parents know what to expect from screening and be prepared to receive the results? Is documentation sent alone so as not to be mixed in or lost amongst other notifications?
2. Is information about weight screening and weight notification communicated to parents and/or children in good time before the process begins allowing for the option to give consent or opt out?
3. Are parents provided with information about how to correctly read and interpret the screening results?
4. Are children provided with a clear explanation of the screening process, who is doing the screening and what the results mean?
5. Do health workers intervene early and provide parents with and help them understand, discuss and approach weight screening results in a way tailored to their needs? Do they have open, respectful discussions with parents in a caring, sensitive and non-judgemental way? Give clear answers to parents’ questions? Provide a supportive environment for decision-making and aid in creating a follow-up plan?
6. When deciding on the mode of notification and the weighing process, have issues of privacy, confidentiality and parent/child preferences been taken into account?
7. Have parent/child preferences been taken into consideration when developing the content, format, presentation, literacy level, terminology and tone of the weight notifications? Is the information provided in a simple, easy to understand way with visual supports for findings and how to interpret them?
8. Has an attempt been made to provide parents with information and guidance on how to communicate with their children about their weight status or how to change habits?

### Implications for research

While we believe we systematically have addressed the review objectives, we found that there are several relevant ongoing trials. Thus, an updated review of research objective one seems indicated. Related, more research on objective one would be useful because the four studies we identified only represent some types of participants, interventions, and outcomes. For example, none of the studies addressed communication when children are underweight, online feedback, notification tools, or information delivered through digital technologies to portable devices such as smart phones or tablets. Additionally, the follow up times of our included studies were short, and it would be important to learn about long term effects. With regard to the qualitative studies, a larger spread of countries, contexts and participants from a variation of backgrounds would be beneficial as well as insights on the views of children and adolescents, including those who are underweight. We also encourage future studies to provide better reporting of context, sampling, methods, and with regard to qualitative studies, researcher reflexivity.

More research is needed on parents’ and children’s’ preferences around the details of timing, amount, and content of weight notification methods. The findings once placed in the framework show that future effect studies could also look at the impact of the timing of the information to parents, information availability, the amount of information parents and children would like to receive as well as issues related to barriers to addressing weight issues in schools and feelings of self-efficacy. Future effect studies could be linked with process evaluations including qualitative studies on order to explore why the intervention work or not.

## Conclusions

In this systematic review, we found that the format of feedback probably made little or no difference in whether parents attended further treatment or recognised their child as overweight or obese. The format of feedback probably made little or no difference in parents’ reactions to the way the weight notification is given, motivation for lifestyle change, understanding how to reduce the risk of overweight, or taking any action. However, parents receiving feedback with motivational interviewing have somewhat greater satisfaction with the way the healthcare provider supports them.

Based on our synthesis of qualitative studies, we found that parents had clear and varied preferences for the format, timing, content, and amount of information they wanted to receive in relation to both the weighing process and weight notification. They also had clear preferences for how they wanted healthcare providers to interact and communicate with them and their children. Both parents and children often felt that they were not receiving enough information and worried about how their results would be kept private during both the weighting itself and the process of notification. Many parents experienced an emotional response when told about their child’s weight, ranging from positive, disbelief, to negative feelings. Those who reacted with disbelief or negatively were less likely to accept their child’s weight status and/or act upon the notification letter.

Taken together, these results show that it is important that program managers and those working with weight assessment and notification programs take parents’ preferences into account when developing feedback formats, consider the mode of feedback they are going to use and provide parents and children with tailored feedback and personalized follow up once a child is identified as overweight or obese.

## Supplementary information

**Additional file 1.** Search strategies.

**Additional file 2.** Excluded studies from full text screening.

**Additional file 3.** GRADE summary of findings tables.

**Additional file 4.** Evidence profiles.

## Data Availability

The datasets used and/or analysed during the current study are available from the corresponding author on reasonable request.

## Abbreviations

BMI: Body mass index
CASP: Critical appraisal skills programme
CBA: Controlled before after study
EPOC: Cochrane effective practice and organisation of care group
GRADE: The Grading of Recommendations Assessment, Development and Evaluation
GRADE-CERQual: Confidence in the Evidence from Reviews of Qualitative Research
OECD: The Organisation for Economic Co-operation and Development
RCT: Randomised control trial
WHO: The World Health Organization
