# Supplementary material for: Communication of children’s weight status: what is effective and what are the children’s and parents’ experiences and preferences? A mixed methods systematic review
Source: BMC Public Health. 2020 Apr 28;20:574. doi: 10.1186/s12889-020-08682-w (PMC7189728; doi:10.1186/s12889-020-08682-w)
Supplement: Supplementary file 1 — Additional file 1. Search strategies. [file 12889_2020_8682_MOESM1_ESM.docx]

Additional file 1: Search strategies

Database: Ovid MEDLINE® and Epub Ahead of Print, In-Process & Other Non-Indexed Citations and Daily 1946 to October 02, 2018

Date: 03.10.2018. Hits: 2736

#SearchesResults

1exp infants/1075519

2exp child/1789455

3adolescent/1887362

4parents/56302

5School Health Services/16181

6School Nursing/5059

7or/1-63343690

8body weight/181335

9body weight changes/5

10weight gain/28979

11weight loss/32436

12overweight/21054

13obesity/163157

14thinness/5378

15body mass index/112629

16pediatric obesity/5753

17or/8-16432438

187 and 1798904

19feedback/28646

20parental notification/430

21communication/77107

22health communication/1611

23or/19-22107121

2418 and 23236

25((infant or infants or baby or babies or neonate* or neo-nate* or adolescen* or child* or boy or boys or girl or girls or juvenile or juveniles or kid or kids or kindergarten* or minor or minors or pediatric or paediatric or preteen* or pre-teen* or preschool* or pre-school* or prepubescen* or pre-pubescen* or pubescen* or pupil or pupils or schoolage* or school-age* or schoolchild* or school-child* or schooler* or school-student* or teen or teens or teenager* or teen-ager* or toddler* or underage* or under-age* or youngster* or youth or young-people or young-person or young-persons or parent or parents or parental or mother or mothers or maternal or father or fathers or paternal or caregiver* or care-giver* or school-nurse* or (school* adj2 health service*)))).ab.1811

24((infant or infants or baby or babies or neonate* or neo-nate* or adolescen* or child* or boy or boys or girl or girls or juvenile or juveniles or kid or kids or kindergarten* or minor or minors or pediatric or paediatric or preteen* or pre-teen* or preschool* or pre-school* or prepubescen* or pre-pubescen* or pubescen* or pupil or pupils or schoolage* or school-age* or schoolchild* or school-child* or schooler* or school-student* or teen or teens or teenager* or teen-ager* or toddler* or underage* or under-age* or youngster* or youth or young-people or young-person or young-persons or parent or parents or parental or mother or mothers or maternal or father or fathers or paternal or caregiver* or care-giver* or school-nurse* or (school* adj2 health service*)) and ((bmi or body mass index or weight) adj2 screen*)).ti,ab. or ((infant or infants or baby or babies or neonate* or neo-nate* or adolescen* or child* or boy or boys or girl or girls or juvenile or juveniles or kid or kids or kindergarten* or minor or minors or pediatric or paediatric or preteen* or pre-teen* or preschool* or pre-school* or prepubescen* or pre-pubescen* or pubescen* or pupil or pupils or schoolage* or school-age* or schoolchild* or school-child* or schooler* or school-student* or teen or teens or teenager* or teen-ager* or toddler* or underage* or under-age* or youngster* or youth or young-people or young-person or young-persons or parent or parents or parental or mother or mothers or maternal or father or fathers or paternal or caregiver* or care-giver* or school-nurse* or (school* adj2 health service*)) and ((bmi or body mass index or weight) and screen*)).id.97

### 25or/21-241971

26(“0400” or “0451” or “1800” or “2000”).md. [empirical study/ prospective study/ quantitative study/ treatment outcome/clinical trial/]2319187

27Experimental Design/10871

28Between Groups Design/112

29Quantitative Methods/3090

30Quasi Experimental Methods/145

31Experiment Controls/897

32Pretesting/237

33Posttesting/136

34Time Series/1928

35Repeated Measures/664

36(random* or trial or intervention? or effect* or impact? or multicenter or multi center or multicentre or multi centre or controlled or control group? or (before adj5 after) or (pre adj5 post) or ((pretest or pre test) and (posttest or post test)) or quasiexperiment* or quasi experiment* or evaluat* or time series or time point? or repeated measur*).ti,ab.2019364

37Meta Analysis/4248

38Systematic Review.md.19853

39((systematic* adj2 (overview or review* or search*)) or meta-anal* or metaanal* or meta-regression* or meta-review* or umbrella review* or overview of reviews or review of reviews or (evidence* adj2 synth*) or synthesis review*).ti,ab,id.53685

40(review and (pubmed or medline)).ti,ab.14713

41or/26-403098210

4225 and 411721

43(“1600” or “0700” or “0750”).md. or (experience* or interview* or qualitative).tw. [1600 Qualitative Study 0700 Interview 0750 Focus Group]974585

4425 and 43573

45questionnaires/ or surveys/ or survey?.tw.265530

4625 and 45241

4742 or 44 or 461756

48limit 47 to yr=”2000-current”1601

49remove duplicates from 481601

Database: Embase 1974 to 2018 October 2 [OVID]

Date: 03.10.2018. Hits: 1857

#SearchesResults

1*child/84219

2*adolescent/26916

3*”minor (person)”/143

4*infant/12830

5*parent/20127

6*school health nursing/3311

7*school health service/6713

81 or 2 or 3 or 4 or 5 or 6 or 7141786

9*body mass/26265

10*obesity/162915

11*adolescent obesity/1094

12*childhood obesity/6331

13*body weight/29191

14*body weight change/290

15*body weight gain/598

16*body weight loss/1660

179 or 10 or 11 or 12 or 13 or 14 or 15 or 16216426

18exp feedback system/104779

19interpersonal communication/147886

2018 or 19249316

218 and 17 and 2086

22((infant or infants or baby or babies or neonate* or neo-nate* or adolescen* or child* or boy or boys or girl or girls or juvenile or juveniles or kid or kids or kindergarten* or minor or minors or pediatric or paediatric or preteen* or pre-teen* or preschool* or pre-school* or prepubescen* or pre-pubescen* or pubescen* or pupil or pupils or schoolage* or school-age* or schoolchild* or school-child* or schooler* or school-student* or teen or teens or teenager* or teen-ager* or toddler* or underage* or under-age* or youngster* or youth or young-people or young-person or young-persons or parent or parents or parental or mother or mothers or maternal or father or fathers or paternal or caregiver* or care-giver* or school-nurse* or (school* adj2 health service*)) and (BMI or body-mass-index or obes* or overweight* or skinny or thin or thinness or underweight* or weight) and (feedback or notification* or notify* or communicat* or report-card* or (provid* adj2 information*) or inform or informing or tell or tellling or talk or talking or conversation or conversations or discuss* or advice* or advicing or advising)).ti,kw.308

23((((feedback or notification* or notify* or communicat* or report-card* or (provid* adj2 information*) or inform or informing or tell or tellling or talk or talking or conversation or conversations or discuss* or advice* or advicing or advising) adj5 (infant or infants or baby or babies or neonate* or neo-nate* or adolescen* or child* or boy or boys or girl or girls or juvenile or juveniles or kid or kids or kindergarten* or minor or minors or pediatric or paediatric or preteen* or pre-teen* or preschool* or pre-school* or prepubescen* or pre-pubescen* or pubescen* or pupil or pupils or schoolage* or school-age* or schoolchild* or school-child* or schooler* or school-student* or teen or teens or teenager* or teen-ager* or toddler* or underage* or under-age* or youngster* or youth or young-people or young-person or young-persons or parent or parents or parental or mother or mothers or maternal or father or fathers or paternal or caregiver* or care-giver* or school-nurse* or (school* adj2 health service*))) and (BMI or body-mass-index or obes* or overweight* or skinny or thin or thinness or underweight* or weight)) or (((feedback or notification* or notify* or communicat* or report-card* or (provid* adj2 information*) or inform or informing or tell or tellling or talk or talking or conversation or conversations or discuss* or advice* or advicing or advising) adj5 (BMI or body-mass-index or obes* or overweight* or skinny or thin or thinness or underweight* or weight)) and (infant or infants or baby or babies or neonate* or neo-nate* or adolescen* or child* or boy or boys or girl or girls or juvenile or juveniles or kid or kids or kindergarten* or minor or minors or pediatric or paediatric or preteen* or pre-teen* or preschool* or pre-school* or prepubescen* or pre-pubescen* or pubescen* or pupil or pupils or schoolage* or school-age* or schoolchild* or school-child* or schooler* or school-student* or teen or teens or teenager* or teen-ager* or toddler* or underage* or under-age* or youngster* or youth or young-people or young-person or young-persons or parent or parents or parental or mother or mothers or maternal or father or fathers or paternal or caregiver* or care-giver* or school-nurse* or (school* adj2 health service*)))).ab.5728

24((infant or infants or baby or babies or neonate* or neo-nate* or adolescen* or child* or boy or boys or girl or girls or juvenile or juveniles or kid or kids or kindergarten* or minor or minors or pediatric or paediatric or preteen* or pre-teen* or preschool* or pre-school* or prepubescen* or pre-pubescen* or pubescen* or pupil or pupils or schoolage* or school-age* or schoolchild* or school-child* or schooler* or school-student* or teen or teens or teenager* or teen-ager* or toddler* or underage* or under-age* or youngster* or youth or young-people or young-person or young-persons or parent or parents or parental or mother or mothers or maternal or father or fathers or paternal or caregiver* or care-giver* or school-nurse* or (school* adj2 health service*)) and ((bmi or body mass index or weight) adj2 screen*)).ti,ab. or ((infant or infants or baby or babies or neonate* or neo-nate* or adolescen* or child* or boy or boys or girl or girls or juvenile or juveniles or kid or kids or kindergarten* or minor or minors or pediatric or paediatric or preteen* or pre-teen* or preschool* or pre-school* or prepubescen* or pre-pubescen* or pubescen* or pupil or pupils or schoolage* or school-age* or schoolchild* or school-child* or schooler* or school-student* or teen or teens or teenager* or teen-ager* or toddler* or underage* or under-age* or youngster* or youth or young-people or young-person or young-persons or parent or parents or parental or mother or mothers or maternal or father or fathers or paternal or caregiver* or care-giver* or school-nurse* or (school* adj2 health service*)) and ((bmi or body mass index or weight) and screen*)).kw.345

2521 or 22 or 23 or 246227

26Meta Analysis/148702

27Systematic Review/178126

28((systematic* adj2 (overview or review* or search*)) or meta-anal* or metaanal* or meta-regression* or meta-review* or umbrella review* or overview of reviews or review of reviews or (evidence* adj2 synth*) or synthesis review*).ti,ab.294007

29(review and (pubmed or medline)).ti,ab.142027

30Randomized Controlled Trial/514854

31Controlled Clinical Trial/458013

32Quasi Experimental Study/4908

33Pretest Posttest Control Group Design/352

34Time Series Analysis/21272

35Experimental Design/15829

36Multicenter Study/194936

37Pretest Posttest Design/3163

38(random* or trial or intervention? or effect* or impact? or multicenter or multi center or multicentre or multi centre or controlled or control group? or (before adj5 after) or (pre adj5 post) or ((pretest or pre test) and (posttest or post test)) or quasiexperiment* or quasi experiment* or evaluat* or time series or time point? or repeated measur*).ti,ab.12224927

39or/26-3812438538

4025 and 394028

41qualitative research/ or (experience* or qualitative).tw. or interview*.tw.1721807

4225 and 411172

43questionnaire/ or survey?.tw.1113697

4425 and 431208

4540 or 42 or 444727

46exp animals/ or exp invertebrate/ or animal experiment/ or animal model/ or animal tissue/ or animal cell/ or nonhuman/24834914

47human/ or normal human/ or human cell/18881831

4846 not (46 and 47)6003435

49(news or editorial or comment).pt.577416

5045 not (48 or 49)4636

51limit 50 to yr=”2000-current”4352

52limit 51 to embase1896

53remove duplicates from 521857

Database: Cochrane Library [CENTRAL & CDSR]

Dat2: 02.10.2018. Hits: 1468 = 1467 at import.

#1[mh infants]14989

#2[mh child]1403

#3[mh ^adolescent]97405

#4[mh ^parents]1061

#5[mh ^”school health services”]1211

#6[mh ^”school nursing”]76

#7(1-#6)112421

#8[mh ^”body weight”]7625

#9[mh ^”body weight changes”]56

#10[mh ^”weight gain”]2221

#11[mh ^”weight loss”]5122

#12[mh ^overweight]3674

#13[mh ^obesity]9741

#14[mh ^thinness]262

#15[mh ^”body mass index”]9203

#16[mh ^”pediatric obesity”]736

#17or #8-#1625829

#18[mh ^feedback]1215

#19[mh ^”parental notification”]2

#20[mh ^communication]1995

#21[mh ^”health communication”]167

#22or #18-#213339

#23#7 and #17 and #2232

#24((infant or infants or baby or babies or neonate* or neo-nate* or adolescen* or child* or boy or boys or girl or girls or juvenile or juveniles or kid or kids or kindergarten* or minor or minors or pediatric or paediatric or preteen* or pre-teen* or preschool* or pre-school* or prepubescen* or pre-pubescen* or pubescen* or pupil or pupils or schoolage* or school-age* or schoolchild* or school-child* or schooler* or school-student* or teen or teens or teenager* or teen-ager* or toddler* or underage* or under-age* or youngster* or youth or young-people or young-person or young-persons or parent or parents or parental or mother or mothers or maternal or father or fathers or paternal or caregiver* or care-giver* or school-nurse* or (school* NEAR/2 health service*)) and (BMI or body-mass-index or obes* or overweight* or skinny or thin or thinness or underweight* or weight) and (feedback or notification* or notify* or communicat* or report-card* or (provid* NEAR/2 information*) or inform or informing or tell or tellling or talk or talking or conversation or conversations or discuss* or advice* or advicing or advising)):ti,kw225

#25((((feedback or notification* or notify* or communicat* or report-card* or (provid* NEAR/2 information*) or inform or informing or tell or tellling or talk or talking or conversation or conversations or discuss* or advice* or advicing or advising) NEAR/5 (infant or infants or baby or babies or neonate* or neo-nate* or adolescen* or child* or boy or boys or girl or girls or juvenile or juveniles or kid or kids or kindergarten* or minor or minors or pediatric or paediatric or preteen* or pre-teen* or preschool* or pre-school* or prepubescen* or pre-pubescen* or pubescen* or pupil or pupils or schoolage* or school-age* or schoolchild* or school-child* or schooler* or school-student* or teen or teens or teenager* or teen-ager* or toddler* or underage* or under-age* or youngster* or youth or young-people or young-person or young-persons or parent or parents or parental or mother or mothers or maternal or father or fathers or paternal or caregiver* or care-giver* or school-nurse* or (school* NEAR/2 health service*))) and (BMI or body-mass-index or obes* or overweight* or skinny or thin or thinness or underweight* or weight)) or (((feedback or notification* or notify* or communicat* or report-card* or (provid* NEAR/2 information*) or inform or informing or tell or tellling or talk or talking or conversation or conversations or discuss* or advice* or advicing or advising) NEAR/5 (BMI or body-mass-index or obes* or overweight* or skinny or thin or thinness or underweight* or weight)) and (infant or infants or baby or babies or neonate* or neo-nate* or adolescen* or child* or boy or boys or girl or girls or juvenile or juveniles or kid or kids or kindergarten* or minor or minors or pediatric or paediatric or preteen* or pre-teen* or preschool* or pre-school* or prepubescen* or pre-pubescen* or pubescen* or pupil or pupils or schoolage* or school-age* or schoolchild* or school-child* or schooler* or school-student* or teen or teens or teenager* or teen-ager* or toddler* or underage* or under-age* or youngster* or youth or young-people or young-person or young-persons or parent or parents or parental or mother or mothers or maternal or father or fathers or paternal or caregiver* or care-giver* or school-nurse* or (school* NEAR/2 health service*)))):ab.1283

#26((infant or infants or baby or babies or neonate* or neo-nate* or adolescen* or child* or boy or boys or girl or girls or juvenile or juveniles or kid or kids or kindergarten* or minor or minors or pediatric or paediatric or preteen* or pre-teen* or preschool* or pre-school* or prepubescen* or pre-pubescen* or pubescen* or pupil or pupils or schoolage* or school-age* or schoolchild* or school-child* or schooler* or school-student* or teen or teens or teenager* or teen-ager* or toddler* or underage* or under-age* or youngster* or youth or young-people or young-person or young-persons or parent or parents or parental or mother or mothers or maternal or father or fathers or paternal or caregiver* or care-giver* or school-nurse* or (school* NEAR/2 health-service*)) and ((bmi or body-mass-index or weight) NEAR/2 screen*)):ti or ((infant or infants or baby or babies or neonate* or neo-nate* or adolescen* or child* or boy or boys or girl or girls or juvenile or juveniles or kid or kids or kindergarten* or minor or minors or pediatric or paediatric or preteen* or pre-teen* or preschool* or pre-school* or prepubescen* or pre-pubescen* or pubescen* or pupil or pupils or schoolage* or school-age* or schoolchild* or school-child* or schooler* or school-student* or teen or teens or teenager* or teen-ager* or toddler* or underage* or under-age* or youngster* or youth or young-people or young-person or young-persons or parent or parents or parental or mother or mothers or maternal or father or fathers or paternal or caregiver* or care-giver* or school-nurse* or (school* NEAR/2 health-service*)) and ((bmi or body-mass-index or weight) NEAR/2 screen*)):ab or ((infant or infants or baby or babies or neonate* or neo-nate* or adolescen* or child* or boy or boys or girl or girls or juvenile or juveniles or kid or kids or kindergarten* or minor or minors or pediatric or paediatric or preteen* or pre-teen* or preschool* or pre-school* or prepubescen* or pre-pubescen* or pubescen* or pupil or pupils or schoolage* or school-age* or schoolchild* or school-child* or schooler* or school-student* or teen or teens or teenager* or teen-ager* or toddler* or underage* or under-age* or youngster* or youth or young-people or young-person or young-persons or parent or parents or parental or mother or mothers or maternal or father or fathers or paternal or caregiver* or care-giver* or school-nurse* or (school* NEAR/2 health-service*)) and ((bmi or body-mass-index or weight) and screen*)):kw207

#27or #23-#261049 [Limits: with Publication Year from 2000 to 2018, in Trials]

#28or #23-#26420 [Limits: with Cochrane Library publication date from Jan 2000 to Sep 2018, in Cochrane Reviews]

#29#27 or #281468

Database: CINAHL [EBSCO]

Date: 04.10.2018. Hits: 801

#QueryResults

S1(MH “Minors (Legal)”)459

S2(MH “Adolescence”)266,514

S3(MH “Parents”)25,964

S4(MH “School Health Nursing”)7,751

S5(MH “School Health Services”)6,386

S6(MH “Infant”)93,656

S7(MH “Child”)251,304

S8(MH “Child, Preschool”)112,908

S9S1 OR S2 OR S3 OR S4 OR S5 OR S6 OR S7 OR S8484,151

S10(MH “Body Weight”)14,511

S11(MH “Body Weight Changes”)351

S12(MH “Thinness”)999

S13(MH “Weight Gain”)6,742

S14(MH “Weight Loss”)11,942

S15(MH “Obesity+”)48,922

S16S10 OR S11 OR S12 OR S13 OR S14 OR S1571,909

S17(MH “Feedback”)7,617

S18(MH “Parental Notification”)52

S19(MH “Communication+”)167,580

S20S17 OR S18 OR S19167,580

S21S9 AND S16 AND S20359

S22TI ( ((infant or infants or baby or babies or neonate* or neo-nate* or adolescen* or child* or boy or boys or girl or girls or juvenile or juveniles or kid or kids or kindergarten* or minor or minors or pediatric or paediatric or preteen* or pre-teen* or preschool* or pre-school* or prepubescen* or pre-pubescen* or pubescen* or pupil or pupils or schoolage* or school-age* or schoolchild* or school-child* or schooler* or school-student* or teen or teens or teenager* or teen-ager* or toddler* or underage* or under-age* or youngster* or youth or young-people or young-person or young-persons or parent or parents or parental or mother or mothers or maternal or father or fathers or paternal or caregiver* or care-giver* or school-nurse* or (school* N1 health-service*)) and (BMI or body-mass-index or obes* or overweight* or skinny or thin or thinness or underweight* or weight) and (feedback or notification* or notify* or communicat* or report-card* or (provid* N1 information*) or inform or informing or tell or tellling or talk or talking or conversation or conversations or discuss* or advice* or advicing or advising)) ) OR SU ( ((infant or infants or baby or babies or neonate* or neo-nate* or adolescen* or child* or boy or boys or girl or girls or juvenile or juveniles or kid or kids or kindergarten* or minor or minors or pediatric or paediatric or preteen* or pre-teen* or preschool* or pre-school* or prepubescen* or pre-pubescen* or pubescen* or pupil or pupils or schoolage* or school-age* or schoolchild* or school-child* or schooler* or school-student* or teen or teens or teenager* or teen-ager* or toddler* or underage* or under-age* or youngster* or youth or young-people or young-person or young-persons or parent or parents or parental or mother or mothers or maternal or father or fathers or paternal or caregiver* or care-giver* or school-nurse* or (school* N1 health-service*)) and (BMI or body-mass-index or obes* or overweight* or skinny or thin or thinness or underweight* or weight) and (feedback or notification* or notify* or communicat* or report-card* or (provid* N1 information*) or inform or informing or tell or tellling or talk or talking or conversation or conversations or discuss* or advice* or advicing or advising)) )510

S23AB ((((feedback or notification* or notify* or communicat* or report-card* or (provid* N1 information*) or inform or informing or tell or tellling or talk or talking or conversation or conversations or discuss* or advice* or advicing or advising) N4 (infant or infants or baby or babies or neonate* or neo-nate* or adolescen* or child* or boy or boys or girl or girls or juvenile or juveniles or kid or kids or kindergarten* or minor or minors or pediatric or paediatric or preteen* or pre-teen* or preschool* or pre-school* or prepubescen* or pre-pubescen* or pubescen* or pupil or pupils or schoolage* or school-age* or schoolchild* or school-child* or schooler* or school-student* or teen or teens or teenager* or teen-ager* or toddler* or underage* or under-age* or youngster* or youth or young-people or young-person or young-persons or parent or parents or parental or mother or mothers or maternal or father or fathers or paternal or caregiver* or care-giver* or school-nurse* or (school* N1 health-service*))) and (BMI or body-mass-index or obes* or overweight* or skinny or thin or thinness or underweight* or weight)) or (((feedback or notification* or notify* or communicat* or report-card* or (provid* N1 information*) or inform or informing or tell or tellling or talk or talking or conversation or conversations or discuss* or advice* or advicing or advising) N4 (BMI or body-mass-index or obes* or overweight* or skinny or thin or thinness or underweight* or weight)) and (infant or infants or baby or babies or neonate* or neo-nate* or adolescen* or child* or boy or boys or girl or girls or juvenile or juveniles or kid or kids or kindergarten* or minor or minors or pediatric or paediatric or preteen* or pre-teen* or preschool* or pre-school* or prepubescen* or pre-pubescen* or pubescen* or pupil or pupils or schoolage* or school-age* or schoolchild* or school-child* or schooler* or school-student* or teen or teens or teenager* or teen-ager* or toddler* or underage* or under-age* or youngster* or youth or young-people or young-person or young-persons or parent or parents or parental or mother or mothers or maternal or father or fathers or paternal or caregiver* or care-giver* or school-nurse* or (school* N1 health-service*))))1,244

S24TI ( ((infant or infants or baby or babies or neonate* or neo-nate* or adolescen* or child* or boy or boys or girl or girls or juvenile or juveniles or kid or kids or kindergarten* or minor or minors or pediatric or paediatric or preteen* or pre-teen* or preschool* or pre-school* or prepubescen* or pre-pubescen* or pubescen* or pupil or pupils or schoolage* or school-age* or schoolchild* or school-child* or schooler* or school-student* or teen or teens or teenager* or teen-ager* or toddler* or underage* or under-age* or youngster* or youth or young-people or young-person or young-persons or parent or parents or parental or mother or mothers or maternal or father or fathers or paternal or caregiver* or care-giver*) and ((bmi or body-mass-index or weight) N1 screen*)) ) OR AB ( ((infant or infants or baby or babies or neonate* or neo-nate* or adolescen* or child* or boy or boys or girl or girls or juvenile or juveniles or kid or kids or kindergarten* or minor or minors or pediatric or paediatric or preteen* or pre-teen* or preschool* or pre-school* or prepubescen* or pre-pubescen* or pubescen* or pupil or pupils or schoolage* or school-age* or schoolchild* or school-child* or schooler* or school-student* or teen or teens or teenager* or teen-ager* or toddler* or underage* or under-age* or youngster* or youth or young-people or young-person or young-persons or parent or parents or parental or mother or mothers or maternal or father or fathers or paternal or caregiver* or care-giver*) and ((bmi or body-mass-index or weight) N1 screen*)) ) OR SU ( ((infant or infants or baby or babies or neonate* or neo-nate* or adolescen* or child* or boy or boys or girl or girls or juvenile or juveniles or kid or kids or kindergarten* or minor or minors or pediatric or paediatric or preteen* or pre-teen* or preschool* or pre-school* or prepubescen* or pre-pubescen* or pubescen* or pupil or pupils or schoolage* or school-age* or schoolchild* or school-child* or schooler* or school-student* or teen or teens or teenager* or teen-ager* or toddler* or underage* or under-age* or youngster* or youth or young-people or young-person or young-persons or parent or parents or parental or mother or mothers or maternal or father or fathers or paternal or caregiver* or care-giver*) and ((bmi or body-mass-index or weight) and screen*)) )1,838

#### S25S21 OR S22 OR S23 OR S243,576

### S26(PT systematic review) OR (MH systematic review) OR (MH meta analysis)71,580

S27TX((systematic* N1 (overview or review* or search*)) or meta-anal* or metaanal* or meta-regression* or meta-review* or umbrella-review* or “overview of reviews” or “review of reviews” or (evidence* N1 synth*) or synthesis-review*)92,283

S28(PT randomized controlled trial) OR (PT clinical trial) OR (PT research) OR (MH randomized controlled trials) OR (MH clinical trials) OR (MH intervention trials) OR (MH nonrandomized trials) OR (MH experimental studies) OR (MH pretest-posttest design+) OR (MH quasi-experimental studies+) OR (MH multicenter studies) OR (MH “Repeated Measures”) OR (MH Controlled Before-After Studies) OR (MH Quantitative Studies) OR (MH Control Group)1,285,462

S29TX (random* or trial or intervention# or effect* or impact# or multicenter or multi-center or multicentre or multi-centre or controlled or control group# or (before N4 after) or (pre N4 post) or ((pretest or pre-test) and (posttest or post-test)) or quasiexperiment* or quasi-experiment* or evaluat* or time-series or time point# or repeated-measur*)1,491,470

#### S30S26 OR S27 OR S28 OR S291,891,019

S31S25 AND S303,055

S32(MH “Interviews”) OR (MH Qualitative Studies)154,595

S33TX (interview* or qualitative or experience* or focus-group*)451,553

S34S25 AND (S32 OR S33)769

S35(MH “Surveys+”)144,751

S36TX survey#210,185

S37S25 AND (S35 OR S36)993

S38S31 OR 34 OR S37 [Limiters - Exclude MEDLINE records; Published Date: 20000101-20181031]801

Database: Centre for Reviews and Dissemniation [DARE & HTA]

Date: 04.10.2018. Hits: 24

1MeSH DESCRIPTOR Infant EXPLODE ALL TREES2964

2MeSH DESCRIPTOR Child EXPLODE ALL TREES4935

3MeSH DESCRIPTOR Adolescent4594

4MeSH DESCRIPTOR Parents170

5MeSH DESCRIPTOR School Health Services159

6MeSH DESCRIPTOR School Nursing8

7#1 OR #2 OR #3 OR #4 OR #5 OR #68699

8MeSH DESCRIPTOR Body weight218

9MeSH DESCRIPTOR Body weight changes0

10MeSH DESCRIPTOR Weight gain155

11MeSH DESCRIPTOR Weight loss464

12MeSH DESCRIPTOR Overweight172

13MeSH DESCRIPTOR Obesity775

14MeSH DESCRIPTOR Thinness4

15MeSH DESCRIPTOR Body Mass Index363

16MeSH DESCRIPTOR pediatric obesity38

17#8 OR #9 OR #10 OR #11 OR #12 OR #13 OR #14 OR #15 OR #161421

18MeSH DESCRIPTOR Feedback31

19MeSH DESCRIPTOR Parental notification0

20MeSH DESCRIPTOR Communication157

21MeSH DESCRIPTOR Health Communication8

22#18 OR #19 OR #20 OR #21195

23#7 AND #17 AND #221

24(((((feedback or notification* or notify* or communicat* or report-card* or (provid* adj2 information*) or inform or informing or tell or tellling or talk or talking or conversation or conversations or discuss* or advice* or advicing or advising) adj5 (infant or infants or baby or babies or neonate* or neo-nate* or adolescen* or child* or boy or boys or girl or girls or juvenile or juveniles or kid or kids or kindergarten* or minor or minors or pediatric or paediatric or preteen* or pre-teen* or preschool* or pre-school* or prepubescen* or pre-pubescen* or pubescen* or pupil or pupils or schoolage* or school-age* or schoolchild* or school-child* or schooler* or school-student* or teen or teens or teenager* or teen-ager* or toddler* or underage* or under-age* or youngster* or youth or young-people or young-person or young-persons or parent or parents or parental or mother or mothers or maternal or father or fathers or paternal or caregiver* or care-giver* or school-nurse* or (school* adj2 health-service*))) and (BMI or body-mass-index or obes* or overweight* or skinny or thin or thinness or underweight* or weight)) or (((feedback or notification* or notify* or communicat* or report-card* or (provid* adj2 information*) or inform or informing or tell or tellling or talk or talking or conversation or conversations or discuss* or advice* or advicing or advising) adj5 (BMI or body-mass-index or obes* or overweight* or skinny or thin or thinness or underweight* or weight)) and (infant or infants or baby or babies or neonate* or neo-nate* or adolescen* or child* or boy or boys or girl or girls or juvenile or juveniles or kid or kids or kindergarten* or minor or minors or pediatric or paediatric or preteen* or pre-teen* or preschool* or pre-school* or prepubescen* or pre-pubescen* or pubescen* or pupil or pupils or schoolage* or school-age* or schoolchild* or school-child* or schooler* or school-student* or teen or teens or teenager* or teen-ager* or toddler* or underage* or under-age* or youngster* or youth or young-people or young-person or young-persons or parent or parents or parental or mother or mothers or maternal or father or fathers or paternal or caregiver* or care-giver* or school-nurse* or (school* adj2 health-service*)))))27

25(((infant or infants or baby or babies or neonate* or neo-nate* or adolescen* or child* or boy or boys or girl or girls or juvenile or juveniles or kid or kids or kindergarten* or minor or minors or pediatric or paediatric or preteen* or pre-teen* or preschool* or pre-school* or prepubescen* or pre-pubescen* or pubescen* or pupil or pupils or schoolage* or school-age* or schoolchild* or school-child* or schooler* or school-student* or teen or teens or teenager* or teen-ager* or toddler* or underage* or under-age* or youngster* or youth or young-people or young-person or young-persons or parent or parents or parental or mother or mothers or maternal or father or fathers or paternal or caregiver* or care-giver*) and ((bmi or body-mass-index or weight) adj2 screen*)))4

26#23 OR #24 OR #25 [Publication year: FROM 2000 TO 2018]24

### Database: Web of Science

Date: 04.10.2018. Hits: 3332

# 1(((((“feedback” or notification* or notify* or communicat* or report-card* or (provid* NEAR/1 information*) or “inform” or “informing” or “tell” or “tellling” or “talk” or “talking” or “conversation” or “conversations” or discuss* or advice* or “advicing” or “advising”) NEAR/4 (“infant” or “infants” or “baby” or “babies” or neonate* or neo-nate* or adolescen* or child* or “boy” or “boys” or “girl” or “girls” or “juvenile” or “juveniles” or “kid” or “kids” or kindergarten* or “minor” or “minors” or “pediatric” or “paediatric” or preteen* or pre-teen* or preschool* or pre-school* or prepubescen* or pre-pubescen* or pubescen* or “pupil” or “pupils” or schoolage* or school-age* or schoolchild* or school-child* or schooler* or school-student* or teen or teens or teenager* or teen-ager* or toddler* or underage* or under-age* or youngster* or “youth” or young-people or young-person or young-persons or “parent” or “parents” or “parental” or “mother” or “mothers” or “maternal” or “father” or “fathers” or “paternal” or caregiver* or care-giver* or school-nurse* or (school* NEAR/1 health-service*))) and (BMI or body-mass-index or obes* or overweight* or skinny or thin or thinness or underweight* or weight)) or (((feedback or notification* or notify* or communicat* or report-card* or (provid* NEAR/1 information*) or inform or informing or tell or tellling or talk or talking or conversation or conversations or discuss* or advice* or advicing or advising) NEAR/4 (“BMI” or “body-mass-index” or obes* or overweight* or “skinny” or “thin” or “thinness” or underweight* or “weight”)) and (“infant” or “infants” or “baby” or “babies” or neonate* or neo-nate* or adolescen* or child* or “boy” or “boys” or “girl” or “girls” or “juvenile” or “juveniles” or “kid” or “kids” or kindergarten* or “minor” or “minors” or “pediatric” or “paediatric” or preteen* or pre-teen* or preschool* or pre-school* or prepubescen* or pre-pubescen* or pubescen* or “pupil” or “pupils” or schoolage* or school-age* or schoolchild* or school-child* or schooler* or school-student* or teen or teens or teenager* or teen-ager* or toddler* or underage* or under-age* or youngster* or “youth” or young-people or young-person or young-persons or “parent” or “parents” or “parental” or “mother” or “mothers” or “maternal” or “father” or “fathers” or “paternal” or caregiver* or care-giver* or school-nurse* or (school* NEAR/1 health-service*)))))4,103

# 2TOPIC: (((“infant” or “infants” or “baby” or “babies” or neonate* or neo-nate* or adolescen* or child* or “boy” or “boys” or “girl” or “girls” or “juvenile” or “juveniles” or “kid” or “kids” or kindergarten* or “minor” or “minors” or “pediatric” or “paediatric” or preteen* or pre-teen* or preschool* or pre-school* or prepubescen* or pre-pubescen* or pubescen* or “pupil” or “pupils” or schoolage* or school-age* or schoolchild* or school-child* or schooler* or school-student* or teen or teens or teenager* or teen-ager* or toddler* or underage* or under-age* or youngster* or “youth” or young-people or young-person or young-persons or “parent” or “parents” or “parental” or “mother” or “mothers” or “maternal” or “father” or “fathers” or “paternal” or caregiver* or care-giver* or school-nurse* or (school* NEAR/1 health-service*)) and ((“bmi” or “body-mass-index” or “weight”) NEAR/1 screen*)))188

# 3#2 OR #14,258

# 4TOPIC: ((random* or “trial” or intervention$ or effect* or impact$ or “multicenter” or “multi-center” or “multicentre” or “multi-centre” or “controlled” or control-group$ or (“before” NEAR/4 “after”) or (“pre” NEAR/4 “post”) or ((“pretest” or “pre-test”) and (“posttest” or “post-test”)) or quasiexperiment* or quasi-experiment* or evaluat* or “time-series” or time-point$ or repeated-measur* or interview* or experience* or “qualitative”))12,115,409

# 5TOPIC: (((systematic* NEAR/1 (overview or review* or search*)) meta-anal* or metaanal* or meta-regression* or meta-review* or umbrella-review* or “overview of reviews” or “review of reviews” or (evidence* NEAR/1 synth*) or synthesis-review*))229,439

# 6TOPIC: ((interview* or qualitative or experience* or focus-group* or survey or surveys))1,985,936

# 7#6 OR #5 OR #412,457,360

# 8#7 AND #33,332[Indexes=SCI-EXPANDED, SSCI Timespan=2000-2018]

### Database: EPISTEMONIKOS

Date: 04.10.2018. Hits: 438

Search 1; hits: 435

(title:((title:(infant OR infants OR baby OR babies OR neonate* OR neo-nate* OR adolescen* OR child* OR boy OR boys OR girl OR girls OR juvenile OR juveniles OR kid OR kids OR kindergarten* OR minOR minORs OR pediatric OR paediatric OR preteen* OR pre-teen* OR preschool* OR pre-school* OR prepubescen* OR pre-pubescen* OR pubescen* OR pupil OR pupils OR schoolage* OR school-age* OR “school age” OR schoolchild* OR school-child* OR “school child” OR “school children” OR schooler* OR “school student” OR “school students” OR teen OR teens OR teenager* OR teen-ager* OR toddler* OR underage* OR under-age* OR youngster* OR youth OR “young people” OR “young person” OR “young persons” OR parent OR parents OR parental OR mother OR mothers OR maternal OR father OR fathers OR paternal OR caregiver* OR care-giver* OR “care giver” OR “care givers” OR “school nurse” OR “school nurses” OR “school health service” OR “school health services”) OR abstract:(infant OR infants OR baby OR babies OR neonate* OR neo-nate* OR adolescen* OR child* OR boy OR boys OR girl OR girls OR juvenile OR juveniles OR kid OR kids OR kindergarten* OR minOR minORs OR pediatric OR paediatric OR preteen* OR pre-teen* OR preschool* OR pre-school* OR prepubescen* OR pre-pubescen* OR pubescen* OR pupil OR pupils OR schoolage* OR school-age* OR “school age” OR schoolchild* OR school-child* OR “school child” OR “school children” OR schooler* OR “school student” OR “school students” OR teen OR teens OR teenager* OR teen-ager* OR toddler* OR underage* OR under-age* OR youngster* OR youth OR “young people” OR “young person” OR “young persons” OR parent OR parents OR parental OR mother OR mothers OR maternal OR father OR fathers OR paternal OR caregiver* OR care-giver* OR “care giver” OR “care givers” OR “school nurse” OR “school nurses” OR “school health service” OR “school health services”)) AND (title:(BMI OR body-mass-index OR “body mass index” OR obes* OR overweight* OR skinny OR thin OR thinness OR underweight* OR weight) OR abstract:(BMI OR body-mass-index OR “body mass index” OR obes* OR overweight* OR skinny OR thin OR thinness OR underweight* OR weight)) AND (title:(feedback OR notification* OR notify* OR communicat* OR repORt-card* OR “repORt card” OR “repORt cards” OR “provide infORmation” OR “providing infORmation” OR infORm OR infORming OR tell OR tellling OR talk OR talking OR conversation OR conversations OR discuss* OR advice* OR advicing OR advising) OR abstract:(feedback OR notification* OR notify* OR communicat* OR repORt-card* OR “repORt card” OR “repORt cards” OR “provide infORmation” OR “providing infORmation” OR infORm OR infORming OR tell OR tellling OR talk OR talking OR conversation OR conversations OR discuss* OR advice* OR advicing OR advising))) OR abstract:((title:(infant OR infants OR baby OR babies OR neonate* OR neo-nate* OR adolescen* OR child* OR boy OR boys OR girl OR girls OR juvenile OR juveniles OR kid OR kids OR kindergarten* OR minOR minORs OR pediatric OR paediatric OR preteen* OR pre-teen* OR preschool* OR pre-school* OR prepubescen* OR pre-pubescen* OR pubescen* OR pupil OR pupils OR schoolage* OR school-age* OR “school age” OR schoolchild* OR school-child* OR “school child” OR “school children” OR schooler* OR “school student” OR “school students” OR teen OR teens OR teenager* OR teen-ager* OR toddler* OR underage* OR under-age* OR youngster* OR youth OR “young people” OR “young person” OR “young persons” OR parent OR parents OR parental OR mother OR mothers OR maternal OR father OR fathers OR paternal OR caregiver* OR care-giver* OR “care giver” OR “care givers” OR “school nurse” OR “school nurses” OR “school health service” OR “school health services”) OR abstract:(infant OR infants OR baby OR babies OR neonate* OR neo-nate* OR adolescen* OR child* OR boy OR boys OR girl OR girls OR juvenile OR juveniles OR kid OR kids OR kindergarten* OR minOR minORs OR pediatric OR paediatric OR preteen* OR pre-teen* OR preschool* OR pre-school* OR prepubescen* OR pre-pubescen* OR pubescen* OR pupil OR pupils OR schoolage* OR school-age* OR “school age” OR schoolchild* OR school-child* OR “school child” OR “school children” OR schooler* OR “school student” OR “school students” OR teen OR teens OR teenager* OR teen-ager* OR toddler* OR underage* OR under-age* OR youngster* OR youth OR “young people” OR “young person” OR “young persons” OR parent OR parents OR parental OR mother OR mothers OR maternal OR father OR fathers OR paternal OR caregiver* OR care-giver* OR “care giver” OR “care givers” OR “school nurse” OR “school nurses” OR “school health service” OR “school health services”)) AND (title:(BMI OR body-mass-index OR “body mass index” OR obes* OR overweight* OR skinny OR thin OR thinness OR underweight* OR weight) OR abstract:(BMI OR body-mass-index OR “body mass index” OR obes* OR overweight* OR skinny OR thin OR thinness OR underweight* OR weight)) AND (title:(feedback OR notification* OR notify* OR communicat* OR repORt-card* OR “repORt card” OR “repORt cards” OR “provide infORmation” OR “providing infORmation” OR infORm OR infORming OR tell OR tellling OR talk OR talking OR conversation OR conversations OR discuss* OR advice* OR advicing OR advising) OR abstract:(feedback OR notification* OR notify* OR communicat* OR repORt-card* OR “repORt card” OR “repORt cards” OR “provide infORmation” OR “providing infORmation” OR infORm OR infORming OR tell OR tellling OR talk OR talking OR conversation OR conversations OR discuss* OR advice* OR advicing OR advising))))

[Publication year: From 2000-2018]

### Search 2; hits: 3

(title:(infant OR infants OR baby OR babies OR neonate* OR neo-nate* OR adolescen* OR child* OR boy OR boys OR girl OR girls OR juvenile OR juveniles OR kid OR kids OR kindergarten* OR minOR minORs OR pediatric OR paediatric OR preteen* OR pre-teen* OR preschool* OR pre-school* OR prepubescen* OR pre-pubescen* OR pubescen* OR pupil OR pupils OR schoolage* OR school-age* OR “school age” OR schoolchild* OR school-child* OR “school child” OR “school children” OR schooler* OR “school student” OR “school students” OR teen OR teens OR teenager* OR teen-ager* OR toddler* OR underage* OR under-age* OR youngster* OR youth OR “young people” OR “young person” OR “young persons” OR parent OR parents OR parental OR mother OR mothers OR maternal OR father OR fathers OR paternal OR caregiver* OR care-giver* OR “care giver” OR “care givers” OR “school nurse” OR “school nurses” OR “school health service” OR “school health services”) OR abstract:(infant OR infants OR baby OR babies OR neonate* OR neo-nate* OR adolescen* OR child* OR boy OR boys OR girl OR girls OR juvenile OR juveniles OR kid OR kids OR kindergarten* OR minOR minORs OR pediatric OR paediatric OR preteen* OR pre-teen* OR preschool* OR pre-school* OR prepubescen* OR pre-pubescen* OR pubescen* OR pupil OR pupils OR schoolage* OR school-age* OR “school age” OR schoolchild* OR school-child* OR “school child” OR “school children” OR schooler* OR “school student” OR “school students” OR teen OR teens OR teenager* OR teen-ager* OR toddler* OR underage* OR under-age* OR youngster* OR youth OR “young people” OR “young person” OR “young persons” OR parent OR parents OR parental OR mother OR mothers OR maternal OR father OR fathers OR paternal OR caregiver* OR care-giver* OR “care giver” OR “care givers” OR “school nurse” OR “school nurses” OR “school health service” OR “school health services”)) AND (title:(bmi-screening OR “bmi screening” OR “body mass index-screening” OR “body-mass-index-screening” OR “body-mass-index screening” OR “weight screening” OR weight-screening) OR abstract:(bmi-screening OR “bmi screening” OR “body mass index-screening” OR “body-mass-index-screening” OR “body-mass-index screening” OR “weight screening” OR weight-screening))

[Publication year: From 2000-2018]
