# Supplementary material for: Communication of children’s weight status: what is effective and what are the children’s and parents’ experiences and preferences? A mixed methods systematic review
Source: BMC Public Health. 2020 Apr 28;20:574. doi: 10.1186/s12889-020-08682-w (PMC7189728; doi:10.1186/s12889-020-08682-w)
Supplement: Supplementary file 2 — Additional file 2. Excluded studies from full text screening. [file 12889_2020_8682_MOESM2_ESM.docx]

Additional file 2: Excluded studies from full text screening

### Effect studies excluded after full text assessment, with reason for exclusion

| **Reference** | **Reason for exclusion** |
| --- | --- |
| Screening, feedback and treatment in overweight 4-8 year old children: the MInT study. <https://www.anzctr.org.au/Trial/Registration/TrialReview.aspx?id=308336&isReview=true> | Trial registration for the MInT study. Included as Dawson 2014. |
| Improving Childhood Obesity-Related Behavior Change Through Better Risk Communication. <https://clinicaltrials.gov/ct2/show/results/NCT03074929> | Trial registration. No protocol published. Estimated study completion September 2020. |
| Almond D, Lee A, Schwartz AE. Impacts of classifying New York City students as overweight. Proceedings of the National Academy of Sciences of the United States of America. 2016;113(13):3488-91. | Not relevant objective. (Weight trajectories of individuals receiving BMI report cards narrowly designated overweight versus narrowly normal BMI.) |
| Avis J, Browne N, Cave A, Fournier R, Haqq A, Holt N, et al. A brief digital health intervention for parents to prevent childhood obesity in primary care: preliminary findings from a randomized controlled trial (RCT). Obesity reviews. 2016;17:142. | Conference abstract. Not relevant intervention. (Primary prevention.) |
| Ariza AJ, Laslo KM, Thomson JS, Seshadri R, Binns HJ, Pediatric Practice Research G. Promoting growth interpretation and lifestyle counseling in primary care. Journal of Pediatrics. 2009;154(4):596-601.e1. | Not relevant intervention. (Practice-directed intervention concerning growth assessment, recognition overweight and counselling.) |
| Banks J, Shield JP, Sharp D. Barriers engaging families and GPs in childhood weight management strategies. British Journal of General Practice. 2011;61(589):e492-7. | Not relevant study design. (No control group.) |
| Bodner ME, Bilheimer A, Gao X, Lyna P, Alexander SC, Dolor RJ, et al. Studying physician-adolescent patient communication in community-based practices: Recruitment challenges and solutions. International Journal of Adolescent Medicine and Health. 2017;29(4):1-8. | Not relevant objective. (Recruitment challenges in Teen CHAT study.) |
| Bonsergent E, Thilly N, Legrand K, Agrinier N, Tessier S, Lecomte E, et al. Process evaluation of a school-based overweight and obesity screening strategy in adolescents. Global Health Promotion. 2013;20(2):76-82. | Not relevant study design. (Process evaluation of PRALIMAP trail. No effect study found.) |
| Bravender T, Tulsky JA, Farrell D, Alexander SC, Østbye T, Lyna P, et al. Teen CHAT: development and utilization of a web-based intervention to improve physician communication with adolescents about healthy weight. Patient education and counseling. 2013;93(3):525‐31. | Not relevant objective. (Development of Teen CHAT study.) |
| Carcone AI, Naar-King S, Brogan KE, Albrecht T, Barton E, Foster T, et al. Provider communication behaviors that predict motivation to change in black adolescents with obesity. Journal of Developmental & Behavioral Pediatrics. 2013;34(8):599-608. | Not relevant intervention. (Obesity treatment.) |
| Costa Jacobsohn G. Information provision, informational value, and relational support: Assessing perceptions of pediatric family-centered communication as predictors of weight-related outcomes in preschool children. Dissertation Abstracts International Section A: Humanities and Social Sciences. 2015;76(6):No Pagination Specified. | Not relevant intervention. (No weight screening.) |
| Davies E. Childhood obesity after NCMP feedback. The delivery of proactive school nurse interventions. Journal of Family Health. 2016;26(4):33-7. | Not primary research. |
| Dennison BA, Nicholas J, de Long R, Prokorym M, Brissette I. Randomized controlled trial of a mailed toolkit to increase use of body mass index percentiles to screen for childhood obesity. Preventing chronic disease. 2009;6(4):A122. | Not relevant outcomes. (Outcomes on physicians’ behaviours.) |
| Dera-de Bie E, Gerver WJ, Jansen M. Training program for overweight prevention in the child’s first year: compilation and results. Nursing & Health Sciences. 2013;15(3):387-97. | Not relevant study design. (Development of training program. No later effect study found.) |
| Doorley E, Young C, O’Shea B, Darker C, Hollywood B, O’Rorke C. Is primary prevention of childhood obesity by education at 13-month immunisations feasible and acceptable? Results from a general practice based pilot study. Irish Medical Journal. 2015;108(1):13-5. | Not relevant intervention and study design (Primary prevention. No control group.) |
| Edwards BA, Powell JR, McGaffey A, Wislo VM, Boron E, D’Amico FJ, et al. Fitwits^TM^ Leads to Improved Parental Recognition of Childhood Obesity and Plans to Encourage Change. Journal of the American Board of Family Medicine: JABFM. 2017;30(2):178-88. | Not relevant study design. (No control group. Fitwits tool. Weight screening component unclear.) |
| Flynn MA, Hall K, Noack A, Clovechok S, Enns E, Pivnick J, et al. Promotion of healthy weights at preschool public health vaccination clinics in Calgary: an obesity surveillance program. Can J Public Health. 2005;96(6):421-6. | Not relevant objective. (Acceptability and feasibility study.) |
| Forman SF, Woods ER. BMI report cards: do they make the grade? Current Opinion in Pediatrics. 2009;21(4):429-30. | Not primary research. |
| Gauthier KI. Influencing perception: The impact of a health information technology based tailored intervention on hispanic parental perception of preschooler weight status. Dissertation Abstracts International: Section B: The Sciences and Engineering. 2015;76(5):No Pagination Specified. | Not relevant study design. (No control group. HeartSmartKids study. No effect study found.) |
| Grimmett C, Croker H, Carnell S, Wardle J. Telling parents their child’s weight status: psychological impact of a weight-screening program. Pediatrics. 2008;122(3):e682-8. | Not relevant study design. (No control group.) |
| Islam NY. Mechanisms of motivational interviewing in a parent-focused pediatric obesity intervention. Dissertation Abstracts International: Section B: The Sciences and Engineering. 2018;79(1):No Pagination Specified. | Not relevant objective. (Analyses of language in MI treatment sessions.) |
| Johnson A, Ziolkowski GA. School-based Body Mass Index screening program. Nutrition Today. 2006;41(6):274-9. | Not relevant study design. (No measures before intervention.) |
| Justus MB, Ryan KW, Rockenbach J, Katterapalli C, Card-Higginson P. Lessons learned while implementing a legislated school policy: body mass index assessments among Arkansas’s public school students. Journal of School Health. 2007;77(10):706-13. | Not relevant objective. (Describes process of implementation.) |
| Kubik MY, Story M, Davey C, Dudovitz B, Zuehlke EU. Providing obesity prevention counseling to children during a primary care clinic visit: results from a pilot study. Journal of the American Dietetic Association. 2008;108(11):1902-6. | Not relevant intervention and design (Weight screening component unclear. NRCT with only one site in each group.) |
| Looney SM, Raynor HA. Examining the effect of three low-intensity pediatric obesity interventions: a pilot randomized controlled trial. Clinical pediatrics. 2014;53(14):1367‐74. | Not relevant intervention. (Obesity treatment.) |
| McDonald SW, Ginez HK, Vinturache AE, Tough SC. Maternal perceptions of underweight and overweight for 6-8 years olds from a Canadian cohort: reporting weights, concerns and conversations with healthcare providers. BMJ Open. 2016;6(10):e012094. | Not relevant objective. (Survey of parental weight perceptions and recall of healthcare providers’ concern.) |
| McGaffey AL, Abatemarco DJ, Jewell IK, Fidler SK, Hughes K. Fitwits MDTM: an office-based tool and games for conversations about obesity with 9- to 12-year-old children. Journal of the American Board of Family Medicine: JABFM. 2011;24(6):768-71. | Not relevant study de-sign. (Feasibility study of Fitwits tool. Weight screening component unclear.) |
| Mickens SD. The effects of body mass index screening and reporting on students’ self-esteem and body image. Dissertation Abstracts International Section A: Humanities and Social Sciences. 2007;68(6-A): | Not possible to access full text publication (PhD dissertation.) |
| Ostbye T, Lyna P, Bodner ME, Alexander SC, Coffman C, Tulsky JA, et al. The Effect of Parental Presence on Weight-Related Discussions Between Physicians and Their Overweight Adolescent Patients. Clinical Pediatrics. 2015;54(12):1218-20. | Not relevant objective. (Analyses of language in weight-related discussions in Teen CHAT study.) |
| Perrin EM, Jacobson Vann JC, Benjamin JT, Skinner AC, Wegner S, Ammerman AS. Use of a pediatrician toolkit to address parental perception of children’s weight status, nutrition, and activity behaviors. Academic pediatrics. 2010;10(4):274-81. | Not relevant study design. (No control group.) |
| Petrou I. ‘F’ for ‘Fat’. Contemporary Pediatrics. 2015;32(9):35-7. | Not primary research. |
| Pollak KI, Alexander SC, Ostbye T, Lyna P, Tulsky JA, Dolor RJ, et al. Primary care physicians’ discussions of weight-related topics with overweight and obese adolescents: results from the Teen CHAT Pilot study. Journal of Adolescent Health. 2009;45(2):205-7. | Not relevant objective (Analyses of content in weight-related discussions in Teen CHAT study.) |
| Pollak KI, Coffman CJ, Tulsky JA, Alexander SC, Ostbye T, Farrell D, et al. The Teen CHAT trail: Teaching PCPS MI to improve weight discussions with overweight adolescents. Annals of Behavioral Medicine. 2015;49:S82-S. | Conference abstract. Results presented in Pollak 2016. |
| Pollak KI, Coffman CJ, Tulsky JA, Alexander SC, Østbye T, Farrell D, et al. Teaching Physicians Motivational Interviewing for Discussing Weight With Overweight Adolescents. Journal of adolescent health. 2016;59(1):96‐103. | Not relevant intervention. (Increase physicians’ use of MI behaviours in weight conversations.) |
| Pridham KA, Krolikowski MM, Limbo RK, Paradowski J, Rudd N, Meurer JR, et al. Guiding mothers’ management of health problems of very low birth-weight infants. Public Health Nursing. 2006;23(3):205-15. | Not relevant intervention. (Treatment of low birth-weight infants.) |
| Randle M, Okely AD, Dolnicar S. Communicating with parents of obese children: Which channels are most effective? Health Expectations: An International Journal of Public Participation in Health Care & Health Policy. 2017;20(2):349-60. | Not relevant objective. (Survey of parents’ preferred information sources.) |
| Raynor HA, Osterholt KM, Hart CN, Jelalian E, Vivier P, Wing RR. Efficacy of U.S. paediatric obesity primary care guidelines: two randomized trials. Pediatric Obesity. 2012;7(1):28-38. | Not relevant intervention. (Treatment of overweight/obese.) |
| Resnicow K, McMaster F, Bocian A, Harris D, Zhou Y, Snetselaar L, et al. Motivational interviewing and dietary counseling for obesity in primary care: an RCT. Pediatrics. 2015;135(4):649‐57. | Not relevant intervention. (MI in treatment of overweight/obese.) |
| Sanders LM, Perrin EM, Yin HS, Bronaugh A, Rothman RL. “Greenlight study “: a controlled trial of low-literacy, early childhood obesity prevention. Pediatrics. 2014;133(6):e1724‐e37. | Not relevant intervention. (Early primary prevention.) |
| Schroeder N, Rushovich B, Bartlett E, Sharma S, Gittelsohn J, Caballero B. Early Obesity Prevention: A Randomized Trial of a Practice-Based Intervention in 0-24-Month Infants. Journal of Obesity. 2015;2015:795859. | Not relevant intervention. (Early primary prevention.) |
| Schwartz RP. Motivational interviewing (patient-centered counseling) to address childhood obesity. Pediatric Annals. 2010;39(3):154-8. | Not primary research. |
| Sealy YM, Zarcadoolas C, Dresser M, Wedemeyer L, Short L, Silver L. Using public health detailing and a family-centered ecological approach to promote patient-provider-parent action for reducing childhood obesity. Childhood Obesity. 2012;8(2):132-46. | Not relevant objective. (Clinics’ uptake of “Obesity in Children Action Kit”.) |
| Steele RG, Wu YP, Cushing CC, Jensen CD. Evaluation of child health matters: a web-based tutorial to enhance school nurses’ communications with families about weight-related health. Journal of school nursing. 2013;29(2):151‐60. | Not relevant intervention. (Change school nurses’ communications.) |
| Söderlund LL, Malmsten J, Bendtsen P, Nilsen P. Applying motivational interviewing (MI) in counselling obese and overweight children and parents in Swedish child healthcare. Health Education Journal. 2010;69(4):390-400. | Not relevant outcomes. (Outcomes on child healthcare nurses’ views and behaviours.) |
| Sweat V, Bruzzese JM, Albert S, Pinero DJ, Fierman A, Convit A. The Banishing Obesity and Diabetes in Youth (BODY) Project: description and feasibility of a program to halt obesity-associated disease among urban high school students. Journal of Community Health. 2012;37(2):365-71. | Not relevant study design. (Feasibility study of weight feedback to overweight and obese, further screening and initiation of treatment.) |
| Thompson JW, Card-Higginson P. Arkansas’ experience: statewide surveillance and parental information on the child obesity epidemic. Pediatrics. 2009;124:S73-82 1p. | Not relevant objective. (Implementation of BMI screening and results in one US state.) |
| Thomson T, Hall W, Balneaves L, Wong S. Waiting to be weighed: a pilot study of the effect of delayed newborn weighing on breastfeeding outcomes. Canadian nurse. 2009;105(6):24‐8. | Not relevant intervention. (Breastfeeding intervention.) |
| van Grieken A, Vlasblom E, Wang L, Beltman M, Boere-Boonekamp MM, L’Hoir MP, et al. Personalized Web-Based Advice in Combination With Well-Child Visits to Prevent Overweight in Young Children: cluster Randomized Controlled Trial. Journal of medical internet research. 2017;19(7):e268. | Not relevant intervention. (Early primary prevention.) |
| Wislo VM, McGaffey A, Scopaz KA, D’Amico FJ, Jewell IK, Bridges MW, et al. Fitwits: preparing residency-based physicians to discuss childhood obesity with preteens. Clinical pediatrics. 2013;52(12):1107‐17. | Not relevant objective. (If the Fitwits tool changed physician comfort and competence.) |

### Qualitative studies excluded after full text assessment, with reason for exclusion

| **Reference** | **Reason for**  **exclusion** |
| --- | --- |
| Akselbo I, Ingebrigsten O. M ødre til barn med overvekt — erfaringer og utfordringer. Nordic Nursing Research / Nordisk Sygeplejeforskning. 2015;5(4):453-63. | Wrong topic of interest |
| Appleton J, Laws R, Russell CG, Fowler C, Campbell KJ, Denney-Wilson E. Infant formula feeding practices and the role of advice and support: an exploratory qualitative study. BMC Pediatr. 2018;18(1):12. | Wrong topic of interest |
| Ariza AJ, Laslo KM, Thomson JS, Seshadri R, Binns HJ, Pediatric Practice Research G. Promoting growth interpretation and lifestyle counseling in primary care. Journal of Pediatrics. 2009;154(4):596-601.e1. | Wrong study design |
| Bailey KE. An exploratory study of child obesity concerns among African American children and parents. Dissertation Abstracts International: Section B: The Sciences and Engineering. 2010;71(5-B):3349. | Wrong topic of interest |
| Barlow J, Whitlock S, Hanson S, Davis H, Hunt C, Kirkpatrick S, et al. Preventing obesity at weaning: parental views about the EMPOWER programme. Child: Care, Health & Development. 2010;36(6):843-9. | Wrong topic of interest |
| Barlow SE, Richert M, Baker EA. Putting context in the statistics: paediatricians' experiences discussing obesity during office visits. Child: Care, Health & Development. 2007;33(4):416-23. | Wrong participants |
| Bentley F, Swift JA, Cook R, Redsell SA. "I would rather be told than not know" - A qualitative study exploring parental views on identifying the future risk of childhood overweight and obesity during infancy. BMC Public Health. 2017;17(1):684. | Wrong topic of interest |
| Berry D, Colindres M, Vu MB, Davis LP, Chung G, Lowenstein LM, et al. Latino caregiver's insight into childhood overweight management and relationships with their health care providers. Hispanic Health Care International. 2009;7(1):11-20. | Wrong topic of interest |
| Carcone AI, Naar-King S, Brogan KE, Albrecht T, Barton E, Foster T, et al. Provider communication behaviors that predict motivation to change in black adolescents with obesity. Journal of Developmental & Behavioral Pediatrics. 2013;34(8):599-608. | Wrong topic of interest |
| Cohen ML, Tanofsky-Kraff M, Young-Hyman D, Yanovski JA. Weight and its relationship to adolescent perceptions of their providers (WRAP): a qualitative and quantitative assessment of teen weight-related preferences and concerns. Journal of Adolescent Health. 2005;37(2):163. | Wrong study design |
| Degrange S, Legrand C, Petre B, Scheen A, Guillaume M. Individual and projected representations of obesity management within the triad patient/caregiver/family. [French]. Medecine des Maladies Metaboliques. 2015;9(6):559-65. | Wrong participants |
| Edmunds LD. Parents' perceptions of health professionals' responses when seeking help for their overweight children. Family Practice. 2005;22(3):287-92. | Wrong topic of interest |
| Falbe J, Friedman LE, Sokal-Gutierrez K, Thompson HR, Tantoco NK, Madsen KA. "She Gave Me the Confidence to Open Up": bridging Communication by Promotoras in a Childhood Obesity Intervention for Latino Families. Health education & behavior. 2017;44(5):728‐37. | Wrong topic of interest |
| Fitzgibbon ML, Beech BM. The role of culture in the context of school-based BMI screening. Pediatrics. 2009;124 Suppl 1:S50-62. | Wrong study design |
| Gellar L, Druker S, Osganian SK, Gapinski MA, Lapelle N, Pbert L. Exploratory research to design a school nurse-delivered intervention to treat adolescent overweight and obesity. Journal of Nutrition Education & Behavior. 2012;44(1):46-54. | Wrong topic of interest |
| Grimmett C, Croker H, Carnell S, Wardle J. Telling parents their child's weight status: psychological impact of a weight-screening program. Pediatrics. 2008;122(3):e682-8. | Wrong study design |
| Guo JD, Vann WF, Jr., Lee JY, Roberts MW. Identification of Preferred Healthy Weight Counseling Approaches for Children in the Dental Setting. Journal of Clinical Pediatric Dentistry. 2018;07:07 | Wrong study design |
| Gutzmer K. "So, you're a lean guy": Care provider, parent, and child communication about weight, diet, and physical activity. Dissertation Abstracts International: Section B: The Sciences and Engineering. 2018;79(11-B(E)):No Pagination Specified. | Wrong topic of interest |
| Haugstvedt KT, Graff-Iversen S, Bechensteen B, Hallberg U. Parenting an overweight or obese child: a process of ambivalence. Journal of Child Health Care. 2011;15(1):71-80. | Wrong topic of interest |
| Hernandez RG, Cheng TL, Serwint JR. Parents' healthy weight perceptions and preferences regarding obesity counseling in preschoolers: pediatricians matter. Clinical Pediatrics. 2010;49(8):790-8. | Wrong study design |
| Hirschfeld-Dicker L, Samuel RD, Tiram Vakrat E, Dubnov-Raz G. Preferred weight-related terminology by parents of children with obesity. Acta Paediatrica. 2018;17:17. | Wrong study design |
| Hjelkrem K, Lien N, Wandel M. Perceptions of slimming and healthiness among Norwegian adolescent girls. Journal of Nutrition Education & Behavior. 2013;45(3):196-203. | Wrong topic of interest |
| Islam NY. Mechanisms of motivational interviewing in a parent-focused pediatric obesity intervention. Dissertation Abstracts International: Section B: The Sciences and Engineering. 2018;79(1-B(E)):No Pagination Specified. | Wrong topic of interest |
| Jachyra P, Anagnostou E, Knibbe TJ, Petta C, Cosgrove S, Chen L, et al. Weighty Conversations: Caregivers', Children's, and Clinicians' Perspectives and Experiences of Discussing Weight-Related Topics in Healthcare Consultations. Autism research : Official Journal of the International Society for Autism Research. 2018;01:01. | Wrong setting |
| Johnson SB, Pilkington LL, Lamp C, He J, Deeb LC. Parent reactions to a school-based body mass index screening program. Journal of School Health. 2009;79(5):216-23. | Wrong study design |
| Knierim SD, Moore SL, Raghunath SG, Yun L, Boles RE, Davidson AJ. Home Visitations for Delivering an Early Childhood Obesity Intervention in Denver: Parent and Patient Navigator Perspectives. Maternal & Child Health Journal. 2018;23:23. | No description of data analysis |
| Lakshman R, Landsbaugh JR, Schiff A, Cohn S, Griffin S, Ong KK. Developing a programme for healthy growth and nutrition during infancy: understanding user perspectives. Child: Care, Health & Development. 2012;38(5):675-82. | Wrong topic of interest |
| Laurent JS. A qualitative exploration into parental recognition of overweight and obesity in pre-adolescents: a process of discovery. Journal of Pediatric Health Care. 2014;28(2):121-7. | Wrong topic of interest |
| Lowenstein LM, Perrin EM, Berry D, Vu MB, Pullen Davis L, Cai J, et al. Childhood obesity prevention: fathers' reflections with healthcare providers. Childhood Obesity. 2013;9(2):137-43. | Wrong topic of interest |
| Lupi JL, Haddad MB, Gazmararian JA, Rask KJ. Parental perceptions of family and pediatrician roles in childhood weight management. Journal of Pediatrics. 2014;165(1):99-103.e2. | Wrong topic of interest |
| McGaffey AL, Abatemarco DJ, Jewell IK, Fidler SK, Hughes K. Fitwits MDTM: an office-based tool and games for conversations about obesity with 9- to 12-year-old children. Journal of the American Board of Family Medicine: JABFM. 2011;24(6):768-71. | Wrong participants |
| Mejia de Grubb MC, Salemi JL, Gonzalez SJ, Sanderson M, Zoorob RJ, Mkanta W, et al. Parenting style and perceptions of children's weight among US Hispanics: a qualitative analysis. Health Promotion International. 2018;33(1):132-9. | Wrong topic of interest |
| Moore LC, Harris CV, Bradlyn AS. Exploring the relationship between parental concern and the management of childhood obesity. Maternal & Child Health Journal. 2012;16(4):902-8. | Wrong study design |
| Morenz-Harbinger DL. Collaboration with parents to improve outcomes in young child obesity. Dissertation Abstracts International Section A: Humanities and Social Sciences. 2014;75(4-A(E)):No Pagination Specified. | Wrong topic of interest |
| O'Kane C, Wallace A, Wilson L, Annis A, Ma DWL, Haines J. Family-Based Obesity Prevention: Perceptions of Canadian Parents of Preschool-Age Children. Canadian Journal of Dietetic Practice & Research. 2018;79(1):13-7. | Wrong topic of interest |
| O'Keefe M, Coat S. Consulting parents on childhood obesity and implications for medical student learning. Journal of Paediatrics & Child Health. 2009;45(10):573-6. | Wrong topic of interest |
| Schetzina KE, Dalton WT, 3rd, Lowe EF, Azzazy N, Vonwerssowetz KM, Givens C, et al. Developing a coordinated school health approach to child obesity prevention in rural Appalachia: results of focus groups with teachers, parents, and students. Rural & Remote Health. 2009;9(4):1157. | Wrong topic of interest |
| Sonneville KR, Plegue MA, Nichols LP, Chang T. 236 - Adolescent Perspectives on Clinical Conversations About Weight. Journal of Adolescent Health. 2018;62:S120-S. | Wrong study design |
| Syrad H, Falconer C, Cooke L, Saxena S, Kessel AS, Viner R, et al. Health and happiness is more important than weight': a qualitative investigation of the views of parents receiving written feedback on their child's weight as part of the National Child Measurement Programme. Journal of Human Nutrition & Dietetics. 2015;28(1):47-55. | Wrong topic of interest |
| Taylor RW, Williams SM, Dawson AM, Taylor BJ, Meredith-Jones K, Brown D. What factors influence uptake into family-based obesity treatment after weight screening. Journal of pediatrics. 2013;163(6):1657‐62.e1. | Wrong study design |
| Tchibindat F, Martin-Prevel Y, Kolsteren P, Maire B, Delpeuch F. Bringing together viewpoints of mothers and health workers to enhance monitoring and promotion of growth and development of children: a case study from the Republic of Congo. J Health Popul Nutr. 2004;22(1):59-67. | Wrong topic of interest |
| Turer CB, Mehta M, Durante R, Wazni F, Flores G. Parental perspectives regarding primary-care weight-management strategies for school-age children. Maternal & Child Nutrition. 2016;12(2):326-38. | Wrong topic of interest |

### Full text not available

| **Reference** |
| --- |
| Mickens SD. The effects of body mass index screening and reporting on students' self-esteem and body image. Dissertation Abstracts International Section A: Humanities and Social Sciences. 2007;68(6-A):2346. |
| Sellers KK. Perceptions of mothers of four year old children who are overweight or obese. Dissertation Abstracts International Section A: Humanities and Social Sciences. 2012;72(9-A):3096. |
