# Supplementary material for: Communication of children’s weight status: what is effective and what are the children’s and parents’ experiences and preferences? A mixed methods systematic review
Source: BMC Public Health. 2020 Apr 28;20:574. doi: 10.1186/s12889-020-08682-w (PMC7189728; doi:10.1186/s12889-020-08682-w)
Supplement: Supplementary file 3 — Additional file 3. GRADE summary of findings tables. [file 12889_2020_8682_MOESM3_ESM.docx]

Additional file 3: GRADE summary of findings tables

Comparison 1: Effect of weight screening feedback using motivational interviewing compared to best practice care using “traffic lights”

| **Population**: Parents of children identified as overweight or obese after weight screening. **Country**: New Zealand.  **Intervention**: Weight screening feedback given using motivational interviewing and “traffic lights”  **Comparison**: Weight screening feedback as best practice care using “traffic lights”. | | | | | | | | | | | | | |
| --- | --- | --- | --- | --- | --- | --- | --- | --- | --- | --- | --- | --- | --- |
| **Outcome,**  **follow-up time** | | **Anticipated absolute effects* (95% CI)** | | | | **Relative**  **effect**  **(95% CI)** | | **No. of**  **participants  (Studies)** | | **Quality of evidence (GRADE)** | |  | |
|  |  | Assumed risk with best practice care using “traffic lights” | | Assumed risk with best practice care using “traffic lights” and motivational interviewing | |  |  |  |  |  |  |  |  |
| **Willingness to participate in further treatment of the child** | | | | | | | | | | | | | |
| Attended first intervention session,  time unclear | | 81.3% | | **74.5% (6.8 % lower)**  (17.0% lower to 3.4% higher) | | - | | 196  (1 RCT) | | ⨁⨁⨁◯  MODERATE ^1^ | |  | |
| **Parental recognition of child’s overweight or obesity** | | | | | | | | | | | |  | |
| Recalled BMI category correctly, 2 weeks | | 98% | | 97% | | - | | 144  (1 RCT) | | ⨁⨁⨁◯  MODERATE ^1^ | |  | |
| **Parental perception of the feedback session** | | | | | | | | | | | | |  |
| Perceived support (HCCQ score^#)^,  2 weeks | Score 5.6 | | Score 6.1  Difference p<0.001 | | - | | 251  (1 RCT) | | ⨁⨁⨁◯  MODERATE ^1^ | |  | |  |
| **Parental motivation for lifestyle change (Treatment self-regulation questionnaire (98))** | | | | | | | | | | | | |  |
| Had autonomous motivation,  2 weeks | Baseline score  5.8 (SD 0.9) | | **0.18 higher at follow-up**  (0.01 to 0.25) | | - | | 251  (1 RCT) | | ⨁⨁⨁◯  MODERATE^1^ | |  | |  |
| Had controlled  motivation,  2 weeks | Baseline score  5.8 (SD 0.9) | | **0.10 lower at follow-up**  (-0.10 to 0.08) | | . | | 251  (1 RCT) | | ⨁⨁⨁◯  MODERATE^1^ | |  | |  |
| **Adverse outcomes of the intervention** | | | | | | | | | | | | |  |
| Was upset about the way information was given §, 2 weeks | Score^¤^  1.64 (SD 1.33) | | **1.6 (0.04 lower)**  (-0.33 lower to 0.26 higher) | | **-** | | 244  (1 RCT) | | ⨁⨁⨁◯  MODERATE^1^ | |  | |  |
| 1. Downgraded by 1 level because of imprecision. | | | | | | | | | | | | |  |

CI: Confidence interval; RCT: Randomised, controlled study; SD: Standard deviation.

- The risk in the intervention group (and its 95% confidence interval) is based on the assumed risk in the comparison group and the relative effect of the intervention (and its 95% CI).

# HCCQ score: Health Care Climate Questionnaire. Assesses parental perception of the degree to which the health care worker was autonomy supportive and targets parental response to this person, rather than the information presented. High score represent higher satisfaction. Maximum score 7 (99).

§: Data restricted to the families of overweight or obese children who agreed to attend follow-up interview.

¤ Ranged on a scale from 1 (not at all) to 7 (very true).

Comparison 2: Effect of written feedback letters supplemented with additional resources or follow up compared to standard written weight feedback letters

| **Population**: Parents of children attending weight screening in school.  **Countries**: UK and USA  **Intervention**: Weight status feedback using standard written weight feedback letters.  **Comparison**: Weight status feedback using standard written weight feedback letters supplemented with additional resources or follow up. | | | | | | | | | | | |
| --- | --- | --- | --- | --- | --- | --- | --- | --- | --- | --- | --- |
| **Outcome,**  **follow-up** | **Anticipated absolute effects (95% CI)** | | | | | | **Relative effect (95% CI)** | **No. of participants  (studies)** | | **Quality of evidence (GRADE)** |  |
|  | Assumed risk with standard written feedback letter | Risk with written feedback letter + online resources | | | Risk with written feedback letter + call from school nurse | |  |  |  |  |  |
| **Parents attended follow up session/contacted health care provider** | | | | | | | | | | |  |
| Contacted health care provider, 4-6 weeks | Not reported | | Not reported |  | | | OR 0.80  (0.59 to 1.10) | 1469  (1 RCT)  (Bailey-  Davies) | | ⨁⨁◯◯  LOW^1^ |  |
| **Parental recognition of child’s overweight or obesity** | | | | | | | | | | |  |
| Classified child’s status correctly, 1 month | Change from baseline 10%  (-7.4% to 27%) | |  | Change from baseline 32% (20% to 44%) | | | - | 105*  (1 CBA)  (Falconer) | | ⨁◯◯◯  VERY LOW^2^ |  |
| Recognised the risks of obesity, 1 month | Change from  baseline -7.9%  (-27% to 11%) | |  | Change from baseline 13%  (-0.5% to 26%) | | | - | 105*  (1 CBA)  (Falconer) | | ⨁◯◯◯  VERY LOW^2^ |  |
| **Parental perception of the information/resources given** | | | | | | | | | | |  |
| Perceived it was useful weight status information, 4-6 weeks | Not reported | | Not reported |  | | | OR 1.05  (0.17 to 6.38) | 1469  (1 RCT)  (Bailey-  Davies) | | ⨁⨁◯◯  LOW^1^ |  |
| Perceived it helped understand weight status, 4-6 weeks | Not reported | | Not reported |  | | | OR 0.84  (0.65 to 1.09) | 1469  (1 RCT)  (Bailey-  Davies) | | ⨁⨁⨁◯  MODERATE ^3^ |  |
| Perceived it helped reduce overweight risk, 4-6 weeks | Not reported | | Not reported |  | |  | OR 1.53  (0.96 to 2.46) | | 1469  (1 RCT)  (Bailey-Davies) | ⨁⨁⨁◯  MODERATE ^3^ |  |
| **Adverse outcomes** | | | | | | | | | | |  |
| Any outcome | The studies did not assess any relevant adverse outcomes | | | | | | | | | |  |
| 1. Downgraded by 2 levels because of unclear risk of bias and imprecision.  2. Downgraded by 3 levels due to study design, risk of bias and imprecision  3. Downgraded by 1 level due to unclear risk of bias | | | | | | | | | | |  |

CI: Confidence interval; RCT: Randomised, controlled study; SMD: Standardised mean difference.

*Response in the subsample of parents with children identified as overweight or obese in the screening.

Comparison 3: Effect of different formats (phrasing) of written weight-screening feedback letters

| **Population**: Parents of children attending weight screening in school, the obese and overweight children only.  **Countries**: Mexico  **Intervention**: Weight status feedback using basic written weight feedback letters.  **Comparison**: Weight status feedback using either risk messages or comparing child to BMI distribution in class. | | | | | |
| --- | --- | --- | --- | --- | --- |
| **Outcome,**  **follow-up** | Proportion with simple written feedback letter (95% CI) | Proportion with written feedback letter containing health risk messages (95% CI) | Proportion with written feedback letter and BMI distribution (95% CI) | **No. of participants  (Studies)** | **Quality of evidence (GRADE)** |
| **Parents attended follow up session/contacted health care provider** | | | | | |
| Attended parents’ information meeting,  2 weeks | 19.6%  (12.0% to 27.2%) | 19.9%  (12.1% to 27.7%) | 22.4%  (14.6% to 30.2%) | 824  (1 RCT) | ⨁⨁◯◯  LOW^1^ |
| Took any action,  3 months | 96.3%  (90.4% to 102%) | 96.7%  (90.8% to 103%) | 93.8%  (86.5% to 99.5%) | 465  (1 RCT) | ⨁⨁⨁◯  MODERATE^2^ |
| **Parental recognition of child’s overweight or obesity** | | | | | |
| Classified child’s status correctly,  3 months | 5.9%  (-5.7% to 17.5%) | 38.8%  (25.9% to 50.0%) | 40.8%  (29.6% to 52.0%) | 459  (1 RCT) | ⨁⨁◯◯  LOW^1^ |
| **Child’s subsequent weight status** | | | | | |
| BMI (kg/m^2^),  3 months | 21.5  (21.2 to 21.9) | 21.6  (21.2 to 21.9) | 21.5  (21.1 to 21.8) | 755  (1 RCT) | ⨁⨁⨁◯  MODERATE ^2^ |
| **Adverse outcomes** | | | | | |
| Any outcome | The studies did not assess any relevant adverse outcomes | | | |  |
| 1. Downgraded by 2 levels because of unclear to high risk of bias and imprecision  2. Downgraded by 1 level because of unclear to high risk of bias | | | | | |

RCT: Randomised, controlled study
