# Supplementary material for: Communication of children’s weight status: what is effective and what are the children’s and parents’ experiences and preferences? A mixed methods systematic review
Source: BMC Public Health. 2020 Apr 28;20:574. doi: 10.1186/s12889-020-08682-w (PMC7189728; doi:10.1186/s12889-020-08682-w)
Supplement: Supplementary file 4 — Additional file 4. Evidence profiles. [file 12889_2020_8682_MOESM4_ESM.docx]

Additional file 4: Evidence profiles

**Evidence profiles for effect findings**

We have removed the columns describing anticipated absolute effect as we were not able to calculate this from the included studies.

Table 1: Effect of weight screening feedback using motivational interviewing compared to best practice care using “traffic lights”

| Quality assessment | | | | | | | | | Summary of findings | | | |
| --- | --- | --- | --- | --- | --- | --- | --- | --- | --- | --- | --- | --- |
| No of participants (studies) Follow-up | Risk of bias | Inconsistency | Indirectness | | | Imprecision | Publication bias | Overall quality of evidence | Study event rates (%) | | | Relative effect (95% CI) |
|  |  |  |  |  |  |  |  |  | with best practice care using “traffic | with motivational interviewing | |  |
| **Willingness to participate in further treatment of the child (Attended first group session)** | | | | | | | | | | | | |
| 196  (1 RCT)  Time unclear | Low | None | | None | Serious | | None | ⨁⨁⨁◯  MODERATE | 81.3% | | **74.5% (6.8 % lower)**  (17.0% lower to 3.4% higher) | - |
| **Parental recognition of child’s overweight or obesity (**BMI category recalled correctly) | | | | | | | | | | | | |
| 144  (1 RCT)  2 weeks | Low | None | | None | Serious | | None | ⨁⨁⨁◯  MODERATE | 98% | | 97% | - |
| Quality assessment | | | | | | | | | Summary of findings | | | |
| No of participants (studies) Follow-up | Risk of bias | Inconsistency | Indirectness | | | Imprecision | Publication bias | Overall quality of evidence | Study event rates (%) | | | Relative effect (95% CI) |
|  |  |  |  |  |  |  |  |  | with best practice care using “traffic | with motivational interviewing | |  |
| **Parental perception of the feedback session (**HCCQ score^#^) | | | | | | | | | | | | |
| 251  (1 RCT)  2 weeks | Low | None | | None | Serious | | None | ⨁⨁⨁◯  MODERATE | Score 5.6 | | Score 6.1  Difference p<0.001 | - |
| **Parental motivation for lifestyle change (**Autonomous motivation) | | | | | | | | | | | | |
| 251  (1 RCT)  2 weeks | Low | None | | None | Serious | | None | ⨁⨁⨁◯  MODERATE | Baseline score  5.8 (SD 0.9) | | **0.18 higher at follow-up**  (0.01 to 0.25) | - |
| **Parental motivation for lifestyle change (Controlled motivation)** | | | | | | | | | | | | |
| 251  (1 RCT)  2 weeks | Low | None | | None | Serious | | None | ⨁⨁⨁◯  MODERATE | Baseline score  5.8 (SD 0.9) | | **0.10 lower at follow-up**  (-0.10 to 0.08) | - |
| **Adverse outcomes of the intervention (**Upset about the way information given) | | | | | | | | | | | | |
| 244  (1 RCT)  2 weeks | Low | None | | None | Serious | | None | ⨁⨁⨁◯  MODERATE | Score^¤^  1.64 (SD 1.33) | | **1.6 (0.04 lower)**  (-0.33 lower to 0.26 higher) | - |

Table 2: Effect of written feedback letters supplemented with additional resources or follow up compared to standard written weight feedback letters.

| Quality assessment | | | | | | | Summary of findings | | | |
| --- | --- | --- | --- | --- | --- | --- | --- | --- | --- | --- |
| No of participants (studies) Follow-up | Risk of bias | Inconsistency | Indirectness | Imprecision | Publication bias | Overall quality of evidence | Study event rates (%) | | | Relative effect (95% CI) |
|  |  |  |  |  |  |  | Assumed risk with standard written feedback letter | Risk with written feedback letter + online resources | Risk with written feedback letter + call from school nurse |  |
| **Parents attended follow up session/contacted health care provider (**Contacted health care provider) | | | | | | | | | | |
| 1469  (1 RCT)  (Bailey-Davies)  4-6 weeks | Unclear | None | None | Serious | None | ⨁⨁◯◯  LOW | Not reported | Not reported | - | OR 0.80  (0.59 to 1.10) |
| **Parental recognition of child’s overweight or obesity (**Correct classification child’s status) | | | | | | | | | | |
| 105*  (1 CBA)  (Falconer)  1 month | High | None | None | Serious | None | ⨁◯◯◯  VERY LOW | Change from baseline 10%  (-7.4% to 27%) | - | Change from baseline 32% (20% to 44%) | - |
| **Parental recognition of child’s overweight or obesity (**Recognises the risks of obesity) | | | | | | | | | | |
| 105*  (1 CBA)  (Falconer)  1 month | High | None | None | Serious | None | ⨁◯◯◯  VERY LOW | Change from  baseline -7.9%  (-27% to 11%) | - | Change from baseline 13 %  (-0.5% to 26%) | - |
| **Parental perception of the information/resources given (**Useful weight status information) | | | | | | | | | | |
| 1469  (1 RCT)  (Bailey-Davies)  4-6 weeks | Unclear | None | None | Serious | None | ⨁⨁◯◯  LOW | Not reported | Not reported | - | OR 1.05  (0.17 to 6.38) |
| **Parental perception of the information/resources given (**Helped understand weight status) | | | | | | | | | | |
| 1469  (1 RCT)  (Bailey-Davies)  4-6 weeks | Unclear | None | None | None | None | ⨁⨁⨁◯  MODERATE | Not reported | Not reported | - | OR 0.84  (0.65 to 1.09) |
| **Parental perception of the information/resources given (**Help reduce overweight risk) | | | | | | | | | | |
| 1469  (1 RCT)  (Bailey-Davies)  4-6 weeks | Unclear | None | None | None | None | ⨁⨁⨁◯  MODERATE | Not reported | Not reported | - | OR 1.53  (0.96 to 2.46) |

Table 3: Effect of different formats (phrasing) of written weight-screening feedback letters.

| Quality assessment | | | | | | | Summary of findings | | | |
| --- | --- | --- | --- | --- | --- | --- | --- | --- | --- | --- |
| No of participants (studies) Follow-up | Risk of bias | Inconsistency | Indirectness | Imprecision | Publication bias | Overall quality of evidence | Study event rates (%) | | | Relative effect (95% CI) |
|  |  |  |  |  |  |  | Proportion with simple written feedback letter (95% CI) | Proportion with written feedback letter containing health risk messages (95% CI) | Proportion with written feedback letter and BMI distribution (95% CI) |  |
| **Parents attended follow up session/contacted health care provider-** Attended parents’ information meeting | | | | | | | | | | |
| 824  (1 RCT)  2 weeks | Unclear-high | None | None | Serious | None | ⨁⨁◯◯  LOW | 19.6%  (12.0% to 27.2%) | 19.9%  (12.1% to 27.7%) | 22.4%  (14.6% to 30.2%) | - |
| **Parents attended follow up session/contacted health care provider-** Any action taken | | | | | | | | | | |
| 465  (1 RCT)  3 months | Unclear-high | None | None | None | None | ⨁⨁⨁◯  MODERATE | 96.3%  (90.4% to 102%) | 96.7%  (90.8% to 103%) | 93.8%  (86.5% to 99.5%) | - |
| **Parental recognition of child’s overweight or obesity -** Correct classification of child’s status | | | | | | | | | | |
| 459  (1 RCT)  3 months | Unclear-high | None | None | Serious | None | ⨁⨁◯◯  LOW | 5.9%  (-5.7% to 17.5%) | 38.8%  (25.9% to 50.0%) | 40.8%  (29.6% to 52.0%) | - |
| **Child’s subsequent weight status -** BMI (kg/m^2^) | | | | | | | | | | |
| 755  (1 RCT)  3 months | Unclear-high | None | None | None | None | ⨁⨁⨁◯  MODERATE | 21.5  (21.2 to 21.9) | 21.6  (21.2 to 21.9) | 21.5  (21.1 to 21.8) | - |

**Evidence profiles for qualitative findings**

**Timing of information**

| **Finding 1:** 1- Some parents felt that there was a lack of communication and information about the weighing and notification process. They wanted information about the weighing process before the testing occurred to know what to expect and again before the results were sent home in order to be prepared to receive the letter. They wanted the information to be up to date with recent measurements. | |
| --- | --- |
| **Assessment for each CERQual component** | |
| *Methodological limitations* | Minor concerns due to poor reporting of reflexivity and evidence supporting findings in a few studies |
| *Coherence* | No or very minor concerns |
| *Relevance* | Major concerns as studies from only one context |
| *Adequacy* | No or very minor concerns |
| **Overall CERQual assessment** | |
| *Confidence* | Moderate confidence |
| **Contributing studies** | |
| Study | Context |
| Alba 2018 | USA, Parents of overweight and obese elementary school students, letter sent home from elementary school |
| Ayash 2012 | USA, Parents of children between the ages of 2 to 13 with a BMI above the 85^th^ percentile, face-to-face with pre or post letter preferences in primary care settings |
| Jorda 2017 | USA, parents who had received BMI referrals for their children in first, third or sixth grade and child was over the 95%, letter sent home from elementary school |
| Ruggieri 2013/**2016** | USA, parents of children in grades K-8, letter home from elementary school |
| Schwartz **2010**/2015 | USA, parents of children who had received a letter stating their child was overweight, letter from elementary school |

**Availability of information**

| **Finding 2:** Many parents believed that they should be asked to give consent for weight screening and the option to opt out. They felt that they had not received this information. Due to this, they felt that they had not had the option to give consent or opt out. | |
| --- | --- |
| **Assessment for each GRADE-CERQual component** | |
| *Methodological limitations* | Moderate concerns due to poor reporting of reflexivity and unclear if findings are supported by evidence |
| *Coherence* | No or very minor concerns |
| *Relevance* | Major concerns due to all studies from one context |
| *Adequacy* | Minor concerns due to thin data from one included study |
| **Overall GRADE-CERQual assessment** | |
| *Confidence* | Low confidence |
| **Contributing studies** | |
| Study | Context |
| Harris 2009 | USA, students and parents receiving letters from school |
| Jorda 2017 | USA, parents who had received BMI referrals for their children in first, third or sixth grade and child was over the 95%, letter sent home from elementary school |
| Ruggieri **2013/2016** | USA, parents of children in grades K-8, letter home from elementary school |

| **Finding 3:** Many parents disliked that the information about and permission for testing was sent with other school documents which led to it being lost, not seen or not remembered. Parents wanted follow up information about nutrition and health sent separately from the results letter for the same reason. | |
| --- | --- |
| **Assessment for each GRADE-CERQual component** | |
| *Methodological limitations* | No or very minor concerns |
| *Coherence* | No or very minor concerns |
| *Relevance* | Moderate concerns due to studies from two study contexts |
| *Adequacy* | Major concerns due to thin data from a few studies |
| **Overall GRADE-CERQual assessment** | |
| *Confidence* | Low confidence |
| **Contributing studies** | |
| Study | Context |
| Alba 2018 | USA, Parents of overweight and obese elementary school students, letter sent home from elementary school |
| Jorda 2017 | USA, parents who had received BMI referrals for their children in first, third or sixth grade and child was over the 95%, letter sent home from elementary school |
| Nnyanzi 2016a | England, parents/guardians after they had received their child’s weight results letter, both those with ideal weight and overweight/obese, letter home to parents from elementary school |

| **Finding 4:** A few parents were frustrated that the school did not provide a platform for parents to give feedback on the weighing process and information/notifications about it. | |
| --- | --- |
| **Assessment for each GRADE-CERQual component** | |
| *Methodological limitations* | No or very minor concerns |
| *Coherence* | No or very minor concerns |
| *Relevance* | Major concerns due to limited study contexts |
| *Adequacy* | Major concerns due to thin data |
| **Overall GRADE-CERQual assessment** | |
| *Confidence* | Very low confidence |
| **Contributing studies** | |
| Study | Context |
| Alba 2018 | USA, Parents of overweight and obese elementary school students, letter sent home from elementary school |
| Nnyanzi 2016a | England, parents/guardians after they had received their child’s weight results letter, both those with ideal weight and overweight/obese, letter home to parents from elementary school |

| **Finding 5:** Parents had varied opinions about whether all children should receive weight notification or only those children who fall outside of the healthy range. Parents who believed all children should receive notification were concerned about privacy and confidentiality. Those who believed only those who fall outside of the healthy weight should receive notification were concerned about the cost of sending notifications. | |
| --- | --- |
| **Assessment for each GRADE-CERQual component** | |
| *Methodological limitations* | No or very minor concerns |
| *Coherence* | No or very minor concerns |
| *Relevance* | Major concerns as only one study context included |
| *Adequacy* | Major concerns as thin data from two studies |
| **Overall GRADE-CERQual assessment** | |
| *Confidence* | Low confidence |
| **Contributing studies** | |
| Study | Context |
| Kubik 2007 | USA, parents of elementary school students, exploratory study to find out how parents wanted to be communicated with |
| Schwartz 2010/2015 | USA, parents of children who had received a letter stating their child was overweight, letter from elementary school |

**Amount of information**

| **Finding 6:** Many parents wanted more information about how to interpret the screening results they received in letters and growth charts. Many felt that they had limited knowledge and understanding of how to interpret the results and needed further explanation and assistance. | |
| --- | --- |
| **Assessment for each GRADE-CERQual component** | |
| *Methodological limitations* | Minor concerns due to poor reporting of reflexivity and unclear if findings supported by evidence in some studies |
| *Coherence* | No or very minor concerns |
| *Relevance* | Moderate concerns due to limited settings |
| *Adequacy* | No or very minor concerns |
| **Overall GRADE-CERQual assessment** | |
| *Confidence* | Moderate confidence |
| **Contributing studies** | |
| Study | Context |
| Alba 2018 | USA, Parents of overweight and obese elementary school students, letter sent home from elementary school |
| Ayash 2012 | USA, Parents of children between the ages of 2 to 13 with a BMI above the 85^th^ percentile, face-to-face with pre or post letter preferences in primary care settings |
| Gillison 2014 | United Kingdom, all parents receiving letters informing them that their child was overweight (91^st^ –98^th^ centile) or very overweight (98^th^-100^th^ centile) through the UK National Child Measurement Programme in 2012, through schools |
| Moyer 2014 | USA, parents/caregivers of 8- to 14-year-old obese ( 95^th^ BMI-for-age percentile) children, letter was the main focus but also discussed preferences for face-to-face interactions with health care workers |
| Ruggieri 2013/**2016** | USA, parents of children in grades K-8, letter home from elementary school |
| Schwartz **2010**/2015 | USA, parents of children who had received a letter stating their child was overweight, letter from elementary school |
| Toftemo 2013 | Norway, parents of overweight children aged 2.5–5.5 years, face-to-face meetings with health care workers |
| Valencia 2016 | USA, mothers and caregivers of infants, face-to-face meetings with health care workers to discuss growth charts |
| Woolford 2007 | USA, mothers of preschool children, face-to-face communication with health care workers |

| **Finding 7:** Many children wanted more information about the weighing process before, during and after the process itself. For example, and introduction session and a follow up session. This lack of information can make them feel nervous, terrified or unsure. | |
| --- | --- |
| **Assessment for each GRADE-CERQual component** | |
| *Methodological limitations* | No or very minor concerns |
| *Coherence* | Minor concerns due to some variation in participant experience |
| *Relevance* | Moderate concerns due to limited study context and most participants from one age group of children (10-11 years old) |
| *Adequacy* | Minor concerns due to thin data from one study |
| **Overall GRADE-CERQual assessment** | |
| *Confidence* | Moderate confidence |
| **Contributing studies** | |
| Study | Context |
| Blood 2011 | United Kingdom, Children aged 10-11 undergoing weight screening in an elementary school |
| Nnyanzi 2016 | England, Children who had been weighed at school aged 10-11, letter home to parents from elementary school |
| Shrewsbury 2010 | Australia, adolescents and unrelated parents of adolescents, face-to-face meetings with health care workers |

**Source of information**

| **Finding 8:** Health care providers were a trusted source of information about a child’s weight and could influence parental motivation to address a child’s weight issues. Parents and adolescents felt weight assessments done by health workers were useful, took their advice seriously, and expected that it was their role to inform them about weight issues. They wanted the clinician to approach the weight conversation first in a sensitive, respectful, direct and positive manner using open questions. They wanted health care providers to be proactive in raising the topic, be forthright in their discussions, provide clear messages and in some cases link the child’s excess weight to health risks. They wanted the provider involved in developing a follow-up plan and to share the responsibility for the plan. Some preferred the health care provider and did not want the school involved. | |
| --- | --- |
| **Assessment for each GRADE-CERQual component** | |
| *Methodological limitations* | Minor concerns due to poor reporting of reflexivity and unclear if findings supported by evidence in some studies |
| *Coherence* | No or very minor concerns |
| *Relevance* | Minor concerns due to a majority of studies from one context |
| *Adequacy* | No or very minor concerns |
| **Overall GRADE-CERQual assessment** | |
| *Confidence* | Moderate confidence |
| **Contributing studies** | |
| Study | Context |
| Alba 2018 | USA, Parents of overweight and obese elementary school students, letter sent home from elementary school |
| Ayash 2012 | USA, Parents of children between the ages of 2 to 13 with a BMI above the 85^th^ percentile, face-to-face with pre or post letter preferences in primary care settings |
| Bolling 2009 | USA, parents of children aged 3 to 6 years and between the 85^th^ and 94^th^ percentile body mass index, motivational interviewing |
| Bossick 2017 | USA, teen patients diagnosed as overweight in the last 12 months and mothers, face-to-face interactions in primary care settings |
| Guerrerro 2011 | USA, low-income Spanish speaking Mexican mothers of obese and healthy weight children ages 2–5 years, face-to-face appointments in primary care setting |
| Harris 2009 | USA, students and parents receiving letters from school |
| Jorda 2017 | USA, parents who had received BMI referrals for their children in first, third or sixth grade and child was over the 95%, letter sent home from elementary school |
| Knierim 2015 | USA, the parent or grandparent/primary caregiver of a 2- to 18-year-old primary care patient, face-to-face interactions with health care workers |
| Kubik 2007 | USA, parents of elementary school students, exploratory study to find out how parents wanted to be communicated with |
| McPherson 2018 | Canada, 7–18‐year old’s with and without disabilities and their caregivers., face-to-face conversations with health care workers |
| Moyer 2014 | USA, parents/caregivers of 8- to 14-year-old obese ( 95^th^ BMI-for-age percentile) children, letter was the main focus but also discussed preferences for face-to-face interactions with health care workers |
| Schwartz **2010**/2015 | USA, parents of children who had received a letter stating their child was overweight, letter from elementary school |
| Shrewsbury 2010 | Australia, adolescents and unrelated parents of adolescents, face-to-face meetings with health care workers |
| Toftemo 2013 | Norway, parents of overweight children aged 2.5–5.5 years, face-to-face meetings with health care workers |
| Valencia 2016 | USA, mothers and caregivers of infants, face-to-face meetings with health care workers to discuss growth charts |

| **Finding 9:** Parents wanted health care providers to intervene early and initiate conversations if they were concerned about a child’s weight and customize or tailor the weighing and communication process to each child. | |
| --- | --- |
| **Assessment for each GRADE-CERQual component** | |
| *Methodological limitations* | Moderate concerns due to poor reporting of reflexivity and findings supported by evidence |
| *Coherence* | No or very minor concerns |
| *Relevance* | No or very minor concerns |
| *Adequacy* | Minor concerns due to thin data from two of the included studies |
| **Overall GRADE-CERQual assessment** | |
| *Confidence* | Moderate confidence |
| **Contributing studies** | |
| Study | Context |
| Ayash 2012 | USA, Parents of children between the ages of 2 to 13 with a BMI above the 85^th^ percentile, face-to-face with pre or post letter preferences in primary care settings |
| Bolling 2009 | USA, parents of children aged 3 to 6 years and between the 85^th^ and 94^th^ percentile body mass index, motivational interviewing |
| Bossick 2017 | USA, teen patients diagnosed as overweight in the last 12 months and mothers, face-to-face interactions in primary care settings |
| McPherson 2018 | Canada, 7–18‐year old’s with and without disabilities and their caregivers., face-to-face conversations with health care workers |
| Toftemo 2013 | Norway, parents of overweight children aged 2.5–5.5 years, face-to-face meetings with health care workers |
| Valencia 2016 | USA, mothers and caregivers of infants, face-to-face meetings with health care workers to discuss growth charts |

| **Finding 10:** Parents felt that there were long wait times to see their health care provider and when they were seen that appointments were rushed. | |
| --- | --- |
| **Assessment for each GRADE-CERQual component** | |
| *Methodological limitations* | Moderate concerns due to poor reporting of reflexivity and findings supported by evidence |
| *Coherence* | Minor concerns due to small variations in participant experiences |
| *Relevance* | Major concerns due to studies from only one study context |
| *Adequacy* | Major concerns due to thin data from two studies |
| **Overall GRADE_CERQual assessment** | |
| *Confidence* | Very low confidence |
| **Contributing studies** | |
| Study | Context |
| Bossick 2017 | USA, teen patients diagnosed as overweight in the last 12 months and mothers, face-to-face interactions in primary care settings |
| Valencia 2016 | USA, mothers and caregivers of infants, face-to-face meetings with health care workers to discuss growth charts |

| **Finding 11:** The way that the health care provider reacted to the weight screening letter from the school or discussed the child’s weight led parents to believe or dismiss the screening results. | |
| --- | --- |
| **Assessment for each GRADE_CERQual component** | |
| *Methodological limitations* | Minor concerns due to poor reporting of reflexivity and unclear if findings supported by evidence in some studies |
| *Coherence* | No or very minor concerns |
| *Relevance* | Major concerns due to studies from a single study context |
| *Adequacy* | Minor concerns due to limited number of contributing studies |
| **Overall GRADE-CERQual assessment** | |
| *Confidence* | Low confidence |
| **Contributing studies** | |
| Study | Context |
| Alba 2018 | USA, Parents of overweight and obese elementary school students, letter sent home from elementary school |
| Schwartz **2010/2015** | USA, parents of children who had received a letter stating their child was overweight, letter from elementary school |

| **Finding 12:** Many parents approved of receiving a letter delivered by confidential standard mail to inform of screening results. Many did not approve of sending the letter home with the child. Those who did not approve of the letter wanted a more personal form of information or communication such as a phone call, email or face-to-face meeting. | |
| --- | --- |
| **Assessment for each GRADE-CERQual component** | |
| *Methodological limitations* | Minor concerns due to poor reporting of reflexivity and unclear if findings supported by evidence in some studies |
| *Coherence* | No or very minor concerns |
| *Relevance* | Major concerns due to limited study contexts |
| *Adequacy* | No or very minor concerns |
| **Overall GRADE-CERQual assessment** | |
| *Confidence* | Moderate confidence |
| **Contributing studies** | |
| Study | Context |
| Alba 2018 | USA, Parents of overweight and obese elementary school students, letter sent home from elementary school |
| Ayash 2012 | USA, Parents of children between the ages of 2 to 13 with a BMI above the 85^th^ percentile, face-to-face with pre or post letter preferences in primary care settings |
| Harris 2009 | USA, students and parents receiving letters from school |
| Jorda 2017 | USA, parents who had received BMI referrals for their children in first, third or sixth grade and child was over the 95%, letter sent home from elementary school |
| Kubik 2007 | USA, parents of elementary school students, exploratory study to find out how parents wanted to be communicated with |
| Moyer 2014 | USA, parents/caregivers of 8- to 14-year-old obese ( 95^th^ BMI-for-age percentile) children, letter was the main focus but also discussed preferences for face-to-face interactions with health care workers |
| Ruggieri 2013/**2016** | USA, parents of children in grades K-8, letter home from elementary school |

| **Finding 13:** Secrecy, privacy and confidentiality were important to both children and parents during (conducted in a private and confidential manner)  and after (who has access to the results and how they are delivered to parents)  the weighing process. Participants were concerned with privacy in order to avoid teasing, bullying, embarrassment and stigma and in some case parents wanted to control access to the screening results so that children could not see them. However, some children wanted the social support of their friends while being weighed and measured. | |
| --- | --- |
| **Assessment for each GRADE-CERQual component** | |
| *Methodological limitations* | Minor concerns due to poor reporting of reflexivity and unclear if findings are supported by evidence in some studies |
| *Coherence* | No or very minor concerns |
| *Relevance* | Major concerns due to lack of variation in context |
| *Adequacy* | No or very minor concerns |
| **Overall GRADE-CERQual assessment** | |
| *Confidence* | Moderate confidence |
| **Contributing studies** | |
| Study | Context |
| Alba 2018 | USA, Parents of overweight and obese elementary school students, letter sent home from elementary school |
| Blood 2011 | United Kingdom, Children aged 10-11 undergoing weight screening in an elementary school |
| Harris 2009 | USA, students and parents receiving letters from school |
| Jorda 2017 | USA, parents who had received BMI referrals for their children in first, third or sixth grade and child was over the 95%, letter sent home from elementary school |
| Kubik 2007 | USA, parents of elementary school students, exploratory study to find out how parents wanted to be communicated with |
| Moyer 2014 | USA, parents/caregivers of 8- to 14-year-old obese ( 95^th^ BMI-for-age percentile) children, letter was the main focus but also discussed preferences for face-to-face interactions with health care workers |
| Ruggieri **2013/2016** | USA, parents of children in grades K-8, letter home from elementary school |
| Schwartz **2010**/**2015** | USA, parents of children who had received a letter stating their child was overweight, letter from elementary school |

| **Finding 14:** Many parents wanted more individual follow up and specific, concrete, practical and age appropriate support and guidance for lifestyle changes for instance through additional information, guidance, supplemental materials or referrals to relevant programs. When this was not done, or felt to be lacking, it led to frustration and confusion and was often experienced as a barrier to addressing their child’s weight issue. | |
| --- | --- |
| **Assessment for each GRADE-CERQual component** | |
| *Methodological limitations* | Moderate concerns due to poor reporting of reflexivity and unclear if findings are supported by evidence |
| *Coherence* | Minor concerns due to small variations in participant experiences |
| *Relevance* | Major concerns due to limited study contexts and population group |
| *Adequacy* | No or very minor concerns |
| **Overall GRADE-CERQual assessment** | |
| *Confidence* | Low confidence |
| **Contributing studies** | |
| Study | Context |
| Alba 2018 | USA, Parents of overweight and obese elementary school students, letter sent home from elementary school |
| Ayash 2012 | USA, Parents of children between the ages of 2 to 13 with a BMI above the 85^th^ percentile, face-to-face with pre or post letter preferences in primary care settings |
| Bossick 2017 | USA, teen patients diagnosed as overweight in the last 12 months and mothers, face-to-face interactions in primary care settings |
| Harris 2009 | USA, students and parents receiving letters from school |
| Kubik 2007 | USA, parents of elementary school students, exploratory study to find out how parents wanted to be communicated with |
| Nnyanzi 2016a | England, parents/guardians after they had received their child’s weight results letter, both those with ideal weight and overweight/obese, letter home to parents from elementary school |
| Schwartz **2010/2015** | USA, parents of children who had received a letter stating their child was overweight, letter from elementary school |
| Thompson 2015 | USA, Parents, letter from elementary or middle school |

**Content of information**

| **Finding 15:** Parents had clear preferences for the format, content, presentation, literacy level and tone of the weight notification letters they received. Many felt that the letter lacked necessary information or wanted more information included to help them take to steps to improve their family’s health. Importantly, they wanted a simple, easy to understand, visual explanation of BMI and how to interpret the results. | |
| --- | --- |
| **Assessment for each GRADE-CERQual component** | |
| *Methodological limitations* | Minor concerns due to poor reporting in a few studies |
| *Coherence* | No or very minor concerns |
| *Relevance* | Major concerns due to limited study settings |
| *Adequacy* | No or very minor concerns |
| **Overall GRADE-CERQual assessment** | |
| *Confidence* | Moderate confidence |
| **Contributing studies** | |
| Study | Context |
| Alba 2018 | USA, Parents of overweight and obese elementary school students, letter sent home from elementary school |
| Ayash 2012 | USA, Parents of children between the ages of 2 to 13 with a BMI above the 85^th^ percentile, face-to-face with pre or post letter preferences in primary care settings |
| Gillison 2014 | United Kingdom, all parents receiving letters informing them that their child was overweight (91^st^ –98^th^ centile) or very overweight (98^th^-100^th^ centile) through the UK National Child Measurement Programme in 2012, through schools |
| Harris 2009 | USA, students and parents receiving letters from school |
| Kubik 2007 | USA, parents of elementary school students, exploratory study to find out how parents wanted to be communicated with |
| Nnyanzi 2016a | England, parents/guardians after they had received their child’s weight results letter, both those with ideal weight and overweight/obese, letter home to parents from elementary school |
| Ruggieri 2013/**2016** | USA, parents of children in grades K-8, letter home from elementary school |
| Schwartz **2010**/2015 | USA, parents of children who had received a letter stating their child was overweight, letter from elementary school |
| Thompson 2015 | USA, Parents, letter from elementary or middle school |

| **Finding 16:** Parents had clear preferences for terminology used in letters and by health care providers when discussing/presenting the issue of children’s weight. This choice of terminology could show respect and promote engagement. These clear preferences for the terminology being used included specific words, to avoid judging, insulting or the feeling that parent’s worries were not being taken seriously. If parents felt defensive, judged or offended they sometimes refused to return to the provider. | |
| --- | --- |
| **Assessment for each GRADE-CERQual component** | |
| *Methodological limitations* | Minor concerns due to poor reporting of reflexivity and unclear if findings supported by evidence in some studies |
| *Coherence* | No or very minor concerns |
| *Relevance* | Major concerns due to limited study contexts |
| *Adequacy* | No or very minor concerns |
| **Overall GRADE-CERQual assessment** | |
| *Confidence* | Moderate confidence |
| **Contributing studies** | |
| Study | Context |
| Ayash 2012 | USA, Parents of children between the ages of 2 to 13 with a BMI above the 85^th^ percentile, face-to-face with pre or post letter preferences in primary care settings |
| Bolling 2009 | USA, parents of children aged 3 to 6 years and between the 85^th^ and 94^th^ percentile body mass index, motivational interviewing |
| Jorda 2017 | USA, parents who had received BMI referrals for their children in first, third or sixth grade and child was over the 95%, letter sent home from elementary school |
| Knierim 2015 | USA, the parent or grandparent/primary caregiver of a 2- to 18-year-old primary care patient, face-to-face interactions with health care workers |
| McPherson 2018 | Canada, 7–18‐year old’s with and without disabilities and their caregivers., face-to-face conversations with health care workers |
| Moyer 2014 | USA, parents/caregivers of 8- to 14-year-old obese ( 95^th^ BMI-for-age percentile) children, letter was the main focus but also discussed preferences for face-to-face interactions with health care workers |
| Thompson 2015 | USA, Parents, letter from elementary or middle school |
| Woolford 2007 | USA, mothers of preschool children, face-to-face communication with health care workers |

| **Finding 17:** Language barriers and not having translators limited communication between parents and the health services. When language barriers arose, parents were often given written materials instead of discussing the child’s situation with the provider. This limited communication was a barrier to growth monitoring. | |
| --- | --- |
| **Assessment for each GRADE_CERQual component** | |
| *Methodological limitations* | Minor concerns due to unclear reporting if the findings were supported by evidence |
| *Coherence* | No or very minor concerns |
| *Relevance* | Major concerns due to a single study setting |
| *Adequacy* | Major concerns due to thin data |
| **Overall GRADE-CERQual assessment** | |
| *Confidence* | Very low confidence |
| **Contributing studies** | |
| Study | Context |
| Ayash 2012 | USA, Parents of children between the ages of 2 to 13 with a BMI above the 85^th^ percentile, face-to-face with pre or post letter preferences in primary care settings |

**Influence between the relationship of information, the way it is communicated and action (using the health belief model**

**The Perceived susceptibility of being overweight**

| **Finding 18:** Some parents expected and accepted the results of the BMI letter and were not surprised. However, the majority of parents did not accept the results of the BMI letter. They did not consider their child overweight. They questioned the credibility of the process, the accuracy of BMI measurements, and that the letter varied from the information given by their health care provider. The feedback they were given did not match their perception of their child and the weight report was often discounted.  Many viewed the letter as a judgement or criticism of their parenting. | |
| --- | --- |
| **Assessment for each GRADE-CERQual component** | |
| *Methodological limitations* | Minor concerns due to poor reporting of sampling, data analysis and reflexivity in a small number of studies |
| *Coherence* | No or very minor concerns |
| *Relevance* | Moderate concerns due to limited study settings |
| *Adequacy* | No or very minor concerns |
| **Overall GRADE-CERQual assessment** | |
| *Confidence* | Moderate confidence |
| **Contributing studies** | |
| Study | Context |
| Alba 2018 | USA, Parents of overweight and obese elementary school students, letter sent home from elementary school |
| Gainsbury 2018 | United Kingdom, Parents of 4-5 year olds who had recently received written feedback from the NCMP representing the full spectrum of feedback options (under-, healthy, over- and very overweight), written feedback at the preschool level |
| Gillison 2014 | United Kingdom, all parents receiving letters informing them that their child was overweight (91^st^ –98^th^ centile) or very overweight (98^th^-100^th^ centile) through the UK National Child Measurement Programme in 2012, through schools |
| Harris 2009 | USA, students and parents receiving letters from school |
| Jorda 2017 | USA, parents who had received BMI referrals for their children in first, third or sixth grade and child was over the 95%, letter sent home from elementary school |
| Moyer 2014 | USA, parents/caregivers of 8- to 14-year-old obese ( 95^th^ BMI-for-age percentile) children, letter was the main focus but also discussed preferences for face-to-face interactions with health care workers |
| Nnyanzi 2016a | England, parents/guardians after they had received their child’s weight results letter, both those with ideal weight and overweight/obese, letter home to parents from elementary school |
| Schwartz **2010**/2015 | USA, parents of children who had received a letter stating their child was overweight, letter from elementary school |
| Toftemo 2013 | Norway, parents of overweight children aged 2.5–5.5 years, face-to-face meetings with health care workers |

| **Finding 19:** Children who were overweight often were surprised by the results and entered a phase of denial or shock. They also questioned if the measurements were right as they felt the results must be a mistake. Weight results could cause changes in social structure among children as they started to identify with others who were the same as them. Many children reacted emotionally to learning their weight status. Those who were overweight often reacted with negative emotions or disbelief, which influenced their mental health and well-being and caused worry. Children who were normal weight often reacted with joy and happiness at the results. | |
| --- | --- |
| **Assessment for each GRADE-CERQual component** | |
| *Methodological limitations* | No or very minor concerns |
| *Coherence* | No or very minor concerns |
| *Relevance* | Major concerns due to limited study contexts |
| *Adequacy* | Minor concerns due to very thick data from one study |
| **Overall GRADE-CERQual assessment** | |
| *Confidence* | Very low confidence |
| **Contributing studies** | |
| Study | Context |
| Nnyanzi 2016 | England, parents/guardians after they had received their child’s weight results letter, both those with ideal weight and overweight/obese, letter home to parents from elementary school |
| Schwartz **2010**/2015 | USA, parents of children who had received a letter stating their child was overweight, letter from elementary school |

| **Finding 20:** Many parents participated in an ‘othering’ process when receiving feedback about their child’s weight. This process contributed to the dismissal of overweight feedback received by themselves or their non-othered peers using language to define themselves and separate them from the ‘other’ parents whom they perceived needed to be the target of obesity prevention and that these ’others’ were often not listening. Another group, parents of normal weight children, believed that they were part of the group doing the right thing and viewed other people, especially those whose children were indicated to have weight problems as not doing things correctly. | |
| --- | --- |
| **Assessment for each GRADE-CERQual component** | |
| *Methodological limitations* | No or very minor concerns |
| *Coherence* | No or very minor concerns |
| *Relevance* | Moderate concerns due to limited study settings |
| *Adequacy* | No or very minor concerns |
| **Overall GRADE-CERQual assessment** | |
| *Confidence* | confidence |
| **Contributing studies** | |
| Study | Context |
| Gainsbury 2018 | United Kingdom, Parents of 4-5 year olds who had recently received written feedback from the NCMP representing the full spectrum of feedback options (under-, healthy, over- and very overweight), written feedback at the preschool level |
| Jorda 2017 | USA, parents who had received BMI referrals for their children in first, third or sixth grade and child was over the 95%, letter sent home from elementary school |
| Nnyanzi 2016a | England, parents/guardians after they had received their child’s weight results letter, both those with ideal weight and overweight/obese, letter home to parents from elementary school |

**The perceived barriers of addressing weight issues in the school system**

| **Finding 21:** Parents commented that on one hand the school was doing the BMI measuring but on the other hand, in most cases, was not making changes to facilitate activity and healthier lifestyles for students within the school environment. | |
| --- | --- |
| **Assessment for each GRADE-CERQual component** | |
| *Methodological limitations* | No or very minor concerns |
| *Coherence* | Minor concerns due to one deviant case from a single child |
| *Relevance* | Major concerns due to all studies from one setting |
| *Adequacy* | Moderate concerns due to thinner data |
| **Overall GRADE-CERQual assessment** | |
| *Confidence* | Very low confidence |
| **Contributing studies** | |
| Study | Context |
| Alba 2018 | USA, Parents of overweight and obese elementary school students, letter sent home from elementary school |
| Jorda 2017 | USA, parents who had received BMI referrals for their children in first, third or sixth grade and child was over the 95%, letter sent home from elementary school |
| Ruggieri **2013**/2016 | USA, parents of children in grades K-8, letter home from elementary school |
| Schwartz **2010**/2015 | USA, parents of children who had received a letter stating their child was overweight, letter from elementary school |

**Cues to action**

| **Finding 22:** Many parents had an emotional response to being informed about their child’s weight, who was informing them about their child’s weight and their child’s weight. These varied from positive/neutral, negative, disbelief and more than one emotion. Often parents cycled through the emotions. This reaction was often tied to the child’s weight status with those receiving healthy weight notifications being most positive. A parent’s emotional reaction could influence their perception of the screening program and the school and their motivation to act. | |
| --- | --- |
| **Assessment for each GRADE-CERQual component** | |
| *Methodological limitations* | Minor concerns due to poor reporting of reflexivity and unclear if the findings are supported by evidence in some studies |
| *Coherence* | No or very minor concerns |
| *Relevance* | Moderate concerns due to limited study contexts |
| *Adequacy* | No or very minor concerns |
| **Overall GRADE-CERQual assessment** | |
| *Confidence* | Moderate confidence |
| **Contributing studies** | |
| Study | Context |
| Alba 2018 | USA, Parents of overweight and obese elementary school students, letter sent home from elementary school |
| Gainsbury 2018 | United Kingdom, Parents of 4-5 year olds who had recently received written feedback from the NCMP representing the full spectrum of feedback options (under-, healthy, over- and very overweight), written feedback at the preschool level |
| Gillison 2014 | United Kingdom, all parents receiving letters informing them that their child was overweight (91^st^ –98^th^ centile) or very overweight (98^th^-100^th^ centile) through the UK National Child Measurement Programme in 2012, through schools |
| Harris 2009 | USA, students and parents receiving letters from school |
| Jorda 2017 | USA, parents who had received BMI referrals for their children in first, third or sixth grade and child was over the 95%, letter sent home from elementary school |
| Moyer 2014 | USA, parents/caregivers of 8- to 14-year-old obese ( 95^th^ BMI-for-age percentile) children, letter was the main focus but also discussed preferences for face-to-face interactions with health care workers |
| Nnyanzi 2016a | England, parents/guardians after they had received their child’s weight results letter, both those with ideal weight and overweight/obese, letter home to parents from elementary school |
| Schwartz **2010**/2015 | USA, parents of children who had received a letter stating their child was overweight, letter from elementary school |

| **Finding 23:** In some cases, parents said that receiving the letter about their child’s weight had been a cue to action. Other parents ignored, downplayed or dismissed the letters and took no action and for some their level of concern did not change. A few parents said the letter had no impact as they had already implemented changes in their household before receiving it and continued with these. | |
| --- | --- |
| **Assessment for each GRADE-CERQual component** | |
| *Methodological limitations* | Minor concerns due to poor reporting of methods in one study |
| *Coherence* | No or very minor concerns |
| *Relevance* | Moderate concerns due to limited study contexts |
| *Adequacy* | No or very minor concerns |
| **Overall GRADE-CERQual assessment** | |
| *Confidence* | Moderate confidence |
| **Contributing studies** | |
| Study | Context |
| Alba 2018 | USA, Parents of overweight and obese elementary school students, letter sent home from elementary school |
| Gillison 2014 | United Kingdom, all parents receiving letters informing them that their child was overweight (91^st^ –98^th^ centile) or very overweight (98^th^-100^th^ centile) through the UK National Child Measurement Programme in 2012, through schools |
| Jorda 2017 | USA, parents who had received BMI referrals for their children in first, third or sixth grade and child was over the 95%, letter sent home from elementary school |
| Nnyanzi 2016a | England, parents/guardians after they had received their child’s weight results letter, both those with ideal weight and overweight/obese, letter home to parents from elementary school |
| Schwartz **2010**/2015 | USA, parents of children who had received a letter stating their child was overweight, letter from elementary school |

**Self-Efficacy**

| **Finding 24:** Many parents discussed their struggles with self-efficacy and their ability to make changes at home. Some felt concerned, hopeless and overwhelmed when it came to choosing which changes to make and how to implement them. They mentioned a lack of knowledge, access to services and finances. | |
| --- | --- |
| **Assessment for each GRADE-CERQual component** | |
| *Methodological limitations* | Minor concerns due to poor reporting of methods in one study |
| *Coherence* | No or very minor concerns |
| *Relevance* | Moderate concerns due to limited study contexts |
| *Adequacy* | Moderate concerns due to relatively thin data from two studies |
| **Overall GRADE-CERQual assessment** | |
| *Confidence* | Low confidence |
| **Contributing studies** | |
| Study | Context |
| Ayash 2012 | USA, Parents of children between the ages of 2 to 13 with a BMI above the 85^th^ percentile, face-to-face with pre or post letter preferences in primary care settings |
| Schwartz **2010**/2015 | USA, parents of children who had received a letter stating their child was overweight, letter from elementary school |
| Toftemo 2013 | Norway, parents of overweight children aged 2.5–5.5 years, face-to-face meetings with health care workers |

| **Finding 25:** Many parents felt they lacked knowledge about how to communicate to their children about their weight or changing habits. They found this distressing and it caused fear and frustration. Some parents did not want children to see the letter or hear the results of their screening for fear of causing harm to self-esteem or body image. Other parents still chose to discuss the screening results with their children but feared doing harm. Many parents felt that involving a child in these discussions should be tailored to the child’s age. Parents wanted guidance and kid friendly suggestions for communicating to children about their weight. | |
| --- | --- |
| **Assessment for each GRADE-CERQual component** | |
| *Methodological limitations* | Minor concerns due to poor reporting of reflexivity |
| *Coherence* | Minor concerns due to small variations in participant experiences |
| *Relevance* | No or very minor concerns |
| *Adequacy* | No or very minor concerns |
| **Overall GRADE-CERQual assessment** | |
| *Confidence* | High confidence |
| **Contributing studies** | |
| Study | Context |
| Alba 2018 | USA, Parents of overweight and obese elementary school students, letter sent home from elementary school |
| Bossikck 2017 | USA, teen patients diagnosed as overweight in the last 12 months and mothers, face-to-face interactions in primary care settings |
| Gillison 2014 | United Kingdom, all parents receiving letters informing them that their child was overweight (91^st^ –98^th^ centile) or very overweight (98^th^-100^th^ centile) through the UK National Child Measurement Programme in 2012, through schools |
| Harris 2009 | USA, students and parents receiving letters from school |
| McPherson 2018 | Canada, 7–18‐year old’s with and without disabilities and their caregivers., face-to-face conversations with health care workers |
| Nnyanzi 2016a | England, parents/guardians after they had received their child’s weight results letter, both those with ideal weight and overweight/obese, letter home to parents from elementary school |
| Schwartz **2010**/2015 | USA, parents of children who had received a letter stating their child was overweight, letter from elementary school |
| Shrewsbury 2010 | Australia, adolescents and unrelated parents of adolescents, face-to-face meetings with health care workers |
| Toftemo 2013 | Norway, parents of overweight children aged 2.5–5.5 years, face-to-face meetings with health care workers |

| **Finding 26:** Some children felt that they had limited information about what they could do about their weight situation. They relied on parents and guardians for information about what could be done. | |
| --- | --- |
| **Assessment for each GRADE-CERQual component** | |
| *Methodological limitations* | Minor concerns due to poor reporting of researcher reflexivity |
| *Coherence* | No or very minor concerns |
| *Relevance* | Major concerns due to one study setting and one participant age group (10-11 years old) |
| *Adequacy* | Major concerns due to thin data from one study |
| **Overall GRADE-CERQual assessment** | |
| *Confidence* | Very low confidence |
| **Contributing studies** | |
| Study | Context |
| Nnyanzi 2016 | England, Children who had been weighed at school aged 10-11, letter home to parents from elementary school |
